# Cellular glycan modification by B3GAT1 broadly restricts influenza virus infection

**DOI:** 10.1038/s41467-022-34111-0

**Published:** 2022-10-29

**Authors:** Joseph D. Trimarco, Sarah L. Nelson, Ryan R. Chaparian, Alexandra I. Wells, Nathan B. Murray, Parastoo Azadi, Carolyn B. Coyne, Nicholas S. Heaton

**Affiliations:** 1 Department of Molecular Genetics and Microbiology, Duke University School of Medicine, Durham, NC USA; 2 Department of Pediatrics, Division of Infectious Diseases, UPMC Children’s Hospital of Pittsburgh, Pittsburgh, PA USA; 3 Complex Carbohydrate Research Center, The University of Georgia, Athens, GA USA; 4 Duke Human Vaccine Institute, Duke University School of Medicine, Durham, NC USA

**Keywords:** Influenza virus, Antiviral agents, Glycobiology, Virus-host interactions

## Abstract

Communicable respiratory viral infections pose both epidemic and pandemic threats and broad-spectrum antiviral strategies could improve preparedness for these events. To discover host antiviral restriction factors that may act as suitable targets for the development of host-directed antiviral therapies, we here conduct a whole-genome CRISPR activation screen with influenza B virus (IBV). A top hit from our screen, beta-1,3-glucuronyltransferase 1 (B3GAT1), effectively blocks IBV infection. Subsequent studies reveal that B3GAT1 activity prevents cell surface sialic acid expression. Due to this mechanism of action, B3GAT1 expression broadly restricts infection with viruses that require sialic acid for entry, including Victoria and Yamagata lineage IBVs, H1N1/H3N2 influenza A viruses (IAVs), and the unrelated enterovirus D68. To understand the potential utility of B3GAT1 induction as an antiviral strategy in vivo, we specifically express B3GAT1 in the murine respiratory epithelium and find that overexpression is not only well-tolerated, but also protects female mice from a lethal viral challenge with multiple influenza viruses, including a pandemic-like H1N1 IAV. Thus, B3GAT1 may represent a host-directed broad-spectrum antiviral target with utility against clinically relevant respiratory viruses.

## Abstract (web summary)

Identification of host antiviral restriction factors could provide targets for antiviral therapy. Here, using a genome-wide CRISPR screen, the authors identify the glycosyltransferase B3GAT1 as a host protein which, when ectopically overexpressed, restricts influenza virus infection in vitro and in mice, as well as other viruses relying on sialic acid for entry.

## Introduction

Respiratory viral infections present a major burden to public health and result in significant morbidity and mortality in the global population annually^1,2^. Further, the aerosol transmission of respiratory infections has facilitated global pandemics with influenza A viruses (IAVs) and coronaviruses and the threat of future pandemics from a variant of these (or currently unknown) viruses remains a source of concern^3,4^. While vaccination is generally considered the best strategy to prevent such infections, many known viral strains still lack effective vaccination strategies^5,6^. Additionally, emerging respiratory viruses with unknown correlates of protection can require significant lead times before protective vaccines can be developed and distributed^7^. In the absence of safe and efficacious vaccines, broad-spectrum antiviral preventative and therapeutic strategies would likely represent a front-line defense against infection.

Current FDA-approved antiviral prophylactic and therapeutic compounds in clinical use against common respiratory-transmitted viruses are predominantly direct-acting antivirals^8,9^. These small molecules directly bind a viral protein to prevent its functionality and inhibit viral replication. However, as viruses both require and can be restricted by different host proteins, there is emerging interest in modulating the host cell itself to prevent viral replication. While direct-acting antivirals often lead to the development of resistant viral mutants^10,11^, antiviral strategies targeting the host are thought to potentially offer a higher barrier to resistance^9^. Another potential benefit of host-directed antiviral strategies is a derivative of the observation that many viruses rely on, or are restricted by, the same host pathways;^12–16^ this suggests that at least some host-directed therapeutics may have broad activity against unrelated viral strains. A major current limitation of this approach, however, is that the host targets which could be safely modulated and broadly efficacious for restricting viral infection are incompletely defined.

The development of high-throughput screening approaches has allowed for rapid discovery of both host dependency factors and viral restriction factors^12–32^. While many screens have been conducted, a clear candidate for the development of a host-directed antiviral has remained elusive. High-priority candidates would theoretically be broadly required for (or restrictive of) multiple viruses, possess an ability to be safely modulated in vivo, and block an essential step of the virus replication cycle. In order to define such factors, screening with influenza viruses offers an attractive experimental system, as multiple features of the life cycle are highly conserved with other viruses^33,34^, virus-permissive cell culture models amenable to screening are readily available, and they are among the top causes of severe virus-induced respiratory illness^35^. Our previous CRISPR/dCas9 activation screening with IAV identified a host factor that displayed strong inhibition of infection, but with activity restricted to a subset of influenza viruses^12^. In order to find novel and potentially more broadly acting restriction factors with activity against human viral strains, we decided to perform a screen with a strain of influenza B virus (IBV) as IBVs are almost exclusively human pathogens^36^ and high-throughput IBV screens for viral restriction factors have not been previously reported.

Using the B/Yamagata/16/1988 (Yam/88) strain of IBV, our CRISPR activation screen identified the host glycosyltransferase beta-1,3-glucuronyltransferase 1 (B3GAT1) as a bona fide viral restriction factor. Subsequent mechanistic studies revealed that B3GAT1 likely prevents viral receptor binding by outcompeting host cell sialyltransferases to prevent cell surface expression of the viral sialic acid receptor. As such, B3GAT1 displayed broad restrictive activity against viruses that require sialic acid for entry. These viruses included IBV strains from both the Victoria and Yamagata lineages, antigenically distinct H1N1 and H3N2 IAV strains, and the phylogenetically unrelated endemic enterovirus D68 (EV-D68). We also showed that B3GAT1 can be transiently, and safely, expressed in the murine respiratory tract to confer protection against lethal influenza virus challenges in vivo. These data represent proof-of-concept for the potential efficacy of host-directed antiviral strategies and highlight B3GAT1 as an attractive potential target for the future development of novel antiviral approaches.

## Results

### A genome-wide CRISPR activation screen identifies restriction factors for influenza B virus

**Fig. 1:**
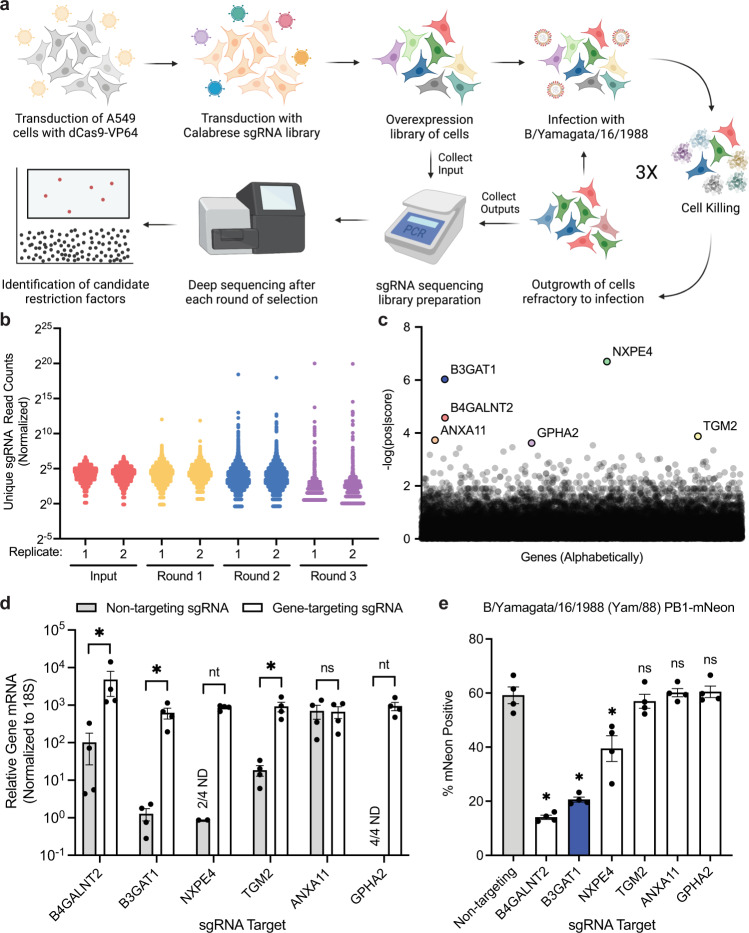
A genome-wide CRISPR activation screen identifies restriction factors for influenza B virus. **a** Schematic of the CRISPR activation screening approach used. sgRNA = single guide RNA. **b** sgRNA read counts detected for each round of infection by B/Yamagata/16/1988 (Yam/88) during the screen. Each point represents a unique sgRNA for each independent screen replicate and sgRNAs not detected are omitted from the graph. **c** Ranked scores for upregulated genes after three rounds of Yam/88 infection. The top six ranked genes are labeled. **d** Expression of the indicated genes in A549 dCas9-VP64 cells after activation with the most enriched sgRNAs from the screen or a non-targeting control sgRNA, measured using qRT-PCR (mean with SEM, *N* = 4 experiments, B4GALNT2 *p = 0.0286, B3GAT1 *p = 0.0286, TGM2 *p = 0.0286, ANXA11 *p* = 0.8857). ND = not detected. **e** Percent of cells infected with Yam/88 PB1-mNeon (MOI = 0.6, 24 HPI, single cycle) in the indicated sgRNA-activated cell lines, measured using flow cytometry (mean with SEM, *N* = 4 experiments, B4GALNT2 *p = 0.0286, B3GAT1 *p = 0.0286, NXPE4 *p = 0.0286, TGM2 *p* = 0.6857, ANXA11 *p* = 0.8857, GPHA2 *p* = 0.6857). Statistical comparisons were all calculated with respect to the non-targeting control. All statistical analyses were performed using a two-tailed Mann-Whitney U test. * indicates *p* < 0.05, ns = not significant, nt = not tested. Source data are provided as a Source Data file.

To discover host factors that may broadly restrict human respiratory viruses, we selected a well-characterized isolate of IBV, Yam/88. To conduct the screen, A549 cells expressing dCas9-VP64 were transduced at low MOI with the Calabrese activation library^37^ of sgRNAs such that each cell received either one or zero sgRNAs. Transduced cells were then selected for antibiotic treatment and passaged for 5 days. Half the library was then collected to determine the input and the remaining cells were infected with Yam/88. After infection, surviving cells were collected and replated. Upon outgrowth, a portion of the cells was collected for sequencing and the remaining library was re-infected with Yam/88. This process was repeated for three rounds of infection until the population became completely resistant to Yam/88 infection. sgRNA libraries were then prepared for sequencing from the genomic DNA isolated from each round of infection and the libraries were sequenced (Fig. 1a).

We reasoned that repeated infection would allow enriched genes to compete internally and ultimately the population would be bottlenecked to consist of a small number of genes with the strongest antiviral activity. As expected, the number of detected sgRNAs decreased over each round of infection, and the unique read counts of a select few sgRNAs became increasingly prominent in the population (Fig. 1b, Supplementary Dataset 1). Bioinformatic analysis using the MAGeCK pipeline^38^ revealed that *NXPE4*, *B3GAT1*, *B4GALNT2*, *TGM2*, *ANXA11*, and *GPHA2* were the six genes most highly ranked in our output population (Fig. 1c, Supplementary Dataset 2). Some, but not all other known restriction factors were also enriched in our screen (e.g., IFITM2, rank #27) indicating that no conclusions as to the biological activity of a host factor should be drawn based on its “absence” amongst our top hits. To perform validation of our most promising screen hits, we individually cloned the highest enriched sgRNA for the top six hits and transduced A549 dCas9-VP64 cells. Upregulation of gene expression upon sgRNA transduction was variable, ranging from no effect to more than 1000-fold induction (Fig. 1d). We then infected these sgRNA-activated cells with a Yam/88 PB1-mNeon reporter virus^39^ and quantified cells infected via flow cytometry. As expected from our previous study^12^, upregulation of B4GALNT2 blocked Yam/88 infection, but in addition, B3GAT1 and NXPE4 also significantly restricted viral infection (Fig. 1e). Since B3GAT1 had not been previously described as a viral restriction factor but had strong restriction activity, we selected B3GAT1 for further characterization.

### B3GAT1 outcompetes sialyltransferases to prevent cell surface sialic acid expression

**Fig. 2:**
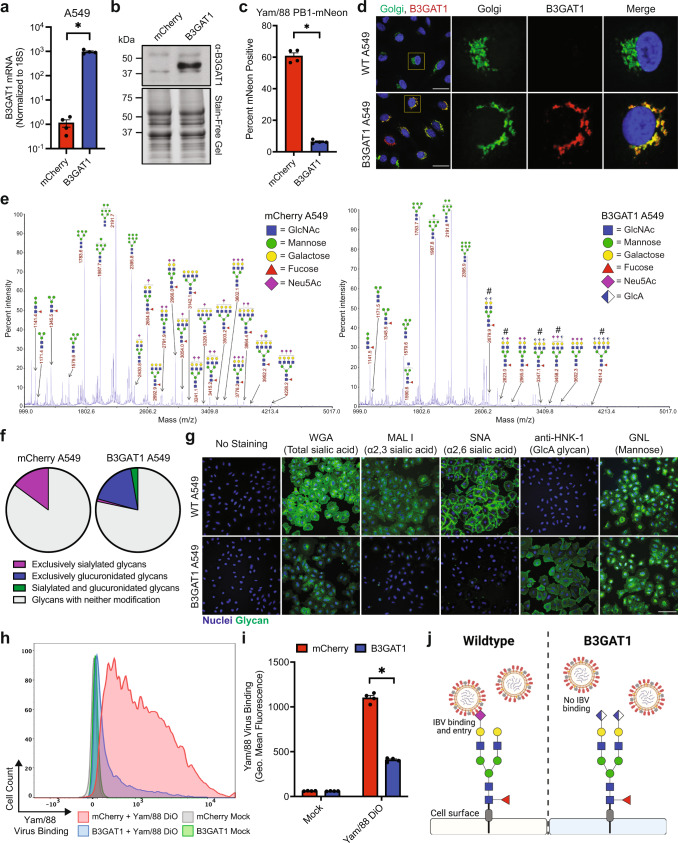
B3GAT1 outcompetes sialyltransferases to prevent cell surface sialic acid expression. **a** qRT-PCR to measure B3GAT1 expression in mCherry control and B3GAT1 transduced A549 cells (mean with SEM, *N* = 4 experiments, *p = 0.0286). **b** Western blot of B3GAT1 protein levels in control or B3GAT1 A549s. Stain-free gel depicts total protein loaded. **c** Percent of control or B3GAT1 A549s infected with Yam/88 PB1-mNeon (MOI = 0.6, 24 HPI, single cycle), measured using flow cytometry (mean with SEM, *N* = 4 experiments, *p = 0.0286). **d** Subcellular localization of B3GAT1 in wild-type (WT) or B3GAT1 A549s. Scale bars are 30 μm. **e** MALDI-TOF mass spectrometry analysis of N-linked glycans released from mCherry- or B3GAT1-overexpressing A549 cells. Unique GlcA-containing glycan signatures are denoted with “#”. **f** Summary of glycan composition determined by MALDI-TOF mass spectrometry for total N-linked glycans released from mCherry- or B3GAT1-overexpressing A549 cells. **g** Fluorescent lectin binding and HNK-1 detection in WT and B3GAT1 A549s. Scale bar is 100 μm. **h** Binding of fluorescently-labeled Yam/88 (Yam/88 DiO) to control or B3GAT1 A549s measured using flow cytometry. Each peak corresponds to one sample, and the flow plot is representative of four replicate experiments. Virus binding is indicated by fluorescence intensity. **i** Geometric mean fluorescence intensity of control or B3GAT1 A549s after incubation with Yam/88 DiO, quantified from flow cytometry data in Fig. 2h (mean with SEM, *N* = 4 experiments, *p = 0.0286). **j** Schematic depicting the mechanism for B3GAT1-mediated restriction of influenza B virus (IBV). All statistical analyses were performed using a two-tailed Mann-Whitney U test. * indicates *p* < 0.05. Source data are provided as a Source Data file.

We were interested in determining the mechanism for B3GAT1-mediated IBV restriction. To facilitate these studies, and to rule out any potential off-target effects of CRISPR activation, we cloned the B3GAT1 ORF and generated a stable overexpression A549 cell line (with a corresponding mCherry-expressing line as a control). B3GAT1 overexpression was confirmed via RNA and protein analysis (Fig. 2a, b). We then infected the lines with Yam/88 PB1-mNeon and observed suppression of viral infection in B3GAT1 A549 cells, as expected (Fig. 2c). B3GAT1 is a glycosyltransferase previously reported to mediate the addition of a glucuronic acid (GlcA) during the biosynthesis of human natural killer 1 (HNK-1) glycans in the secretory pathway^40^ and indeed, we observed a Golgi localization consistent of the protein with that activity (Fig. 2d). To investigate specifically how B3GAT1 activity would alter surface glycan species when ectopically overexpressed, we released total N-linked glycans from mCherry and B3GAT1 A549 cells and subjected them to matrix-assisted laser desorption/ionization time-of-flight (MALDI-TOF) mass spectrometry. We found that while diverse glycans containing sialic acid were abundant in the mCherry A549 cells, many of these species were either not detected or minimally present in B3GAT1 A549 cells (Fig. 2e, f, Supplementary Dataset 3). Additionally, B3GAT1 A549 cells uniquely contained a variety of glucuronidated (GlcA-containing) glycans, while none of these GlcA-containing glycans were detected in the mCherry A549 sample (Fig. 2e, f, Supplementary Dataset 3). This trend was also observable on the monosaccharide level as resolved via gas chromatography-mass spectrometry (GC-MS); B3GAT1 A549 cells contained relatively less Neu5Ac and more GlcA compared to mCherry A549 cells (Supplementary Fig 1).

These data suggested that inhibition of Yam/88 infection was likely due to the replacement of the sialic acid viral receptor with GlcA. We decided to test this hypothesis with fluorescently labeled lectins specific for different terminal sugars. Wheat germ agglutinin (WGA) broadly binds sialic acids and indeed, staining was reduced in B3GAT1 overexpression cells (Fig. 2g). Since different sialic acid linkages (α2,3 vs α2,6) can both serve as receptors for influenza viruses^41,42^, we stained with *Maackia amurensis* lectin I (MAL I) and *Sambucus nigra* lectin (SNA) to show that B3GAT1 expression reduced both α2,3- and α2,6-linked sialic acid expression on the surface of cells, respectively (Fig. 2g). Conversely, an antibody that can recognize a terminal GlcA sugar as part of the HNK-1 epitope failed to stain wild-type A549 cells but did so in the context of B3GAT1 overexpression (Fig. 2g). *Galanthus nivalis* lectin (GNL), which detects the mannose sugars in the glycan, stained both cell lines, as expected (Fig. 2g). Finally, to resolve any potential nonuniform effects of B3GAT1 activity on the small amount of remaining α2,3- and α2,6-linked sialic acids, we subjected per-O-methylated, sialylated, N-linked glycans released from mCherry and B3GAT1 A549 cells to tandem mass spectrometry^43^. These data indicated a slightly higher proportion of α2,6-linked sialic acid in the remaining sialic acid fraction on B3GAT1 A549 cells compared to mCherry A549 cells (Supplementary Fig 2), suggesting B3GAT1 may differentially affect sialic-acid containing glycans. Taken together, however, the data indicate that sialic acids, irrespective of linkages, are broadly reduced in B3GAT1-overexpressing A549 cells (Fig. 2e, f, g, Supplementary Fig 1), likely due to B3GAT1 outcompeting sialyltransferases for immature glycan substrates. This may be partially explained by the apparent localization of overexpressed B3GAT1 throughout Golgi compartments (Fig. 2d), while sialylation of N-linked glycans is generally thought to be a late glycosylation event completed by sialyltransferases localized to the *trans* Golgi and even to post-Golgi compartments^44,45^.

Upon observing that B3GAT1 expression reduces surface sialic acid expression, we hypothesized that the binding of influenza virions to host cells would be impaired and that lack of receptor binding was ultimately responsible for the inhibition of infection. To directly test this, we fluorescently labeled Yam/88 virions then incubated the fluorescent virus with either B3GAT1 or mCherry A549 cells and quantified virus binding to the cell surface via flow cytometry; overexpression of B3GAT1 indeed reduced the ability of Yam/88 to bind to cells (Fig. 2h,i). From these data, we propose a model in which B3GAT1 uniquely outcompetes host sialyltransferases to prevent cell surface expression of sialic acid, which precludes IBV binding and subsequent cell entry (Fig. 2j).

### B3GAT1 broadly restricts viruses that require sialic acid for cell entry

Sialic acid is required for entry into the host cell by many viruses^33^, including the vast majority of influenza viruses. We, therefore, hypothesized that B3GAT1 would restrict any viruses that require sialic acid for entry. To test this, we assembled a panel of diverse influenza viruses representing Yamagata/Victoria-lineage IBVs and H1N1/H3N2 subtype IAVs isolated from 1933 to 2019. We first performed an experiment to understand the range of sialic acids that the selected viruses utilized as receptors. To do this, we treated A549 cells with an α2,3-specific sialidase and then infected with each virus individually. As expected, we observed highly differential abilities of the viruses to cause infection after removal of α2,3-linked sialic acids. (Supplementary Fig 3). We interpreted these data to mean that the affected viruses were mostly dependent on α2,3-linked sialic acids for entry, and those that were largely unaffected were predominantly using α2,6-linked sialic acids (or other glycans) for entry.

**Fig. 3:**
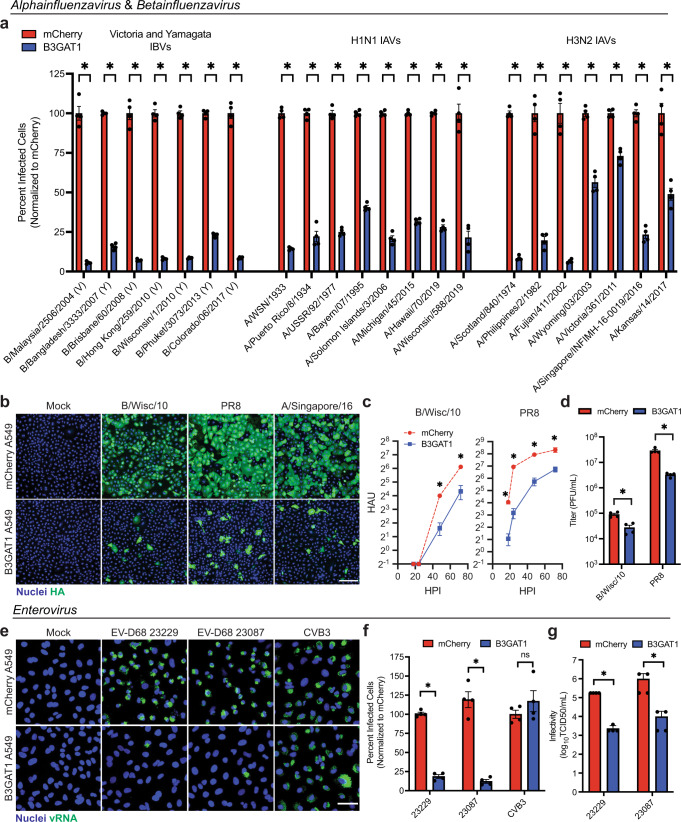
B3GAT1 broadly restricts viruses that require sialic acid for cell entry. **a** Single cycle infections (24 HPI) of control and B3GAT1 A549s with a panel of influenza viruses (mean with SEM, *N* = 4 experiments, *p = 0.0286 for all virus strains). Viruses are ordered based on year of isolation. Percent infection was measured using high-content imaging after staining for the viral HA protein. V = Victoria-lineage IBV, Y = Yamagata-lineage IBV. **b** Representative images for three influenza viruses included in Fig. 3a. B/Wisc/10 = B/Wisconsin/1/2010, PR8 = A/Puerto Rico/8/1934, A/Singapore/16 = A/Singapore/INFIMH-16-0019/2016. Scale bar is 200 µm. **c** Multicycle growth of select influenza strains on control and B3GAT1 MDCKs (MOI B/Wisc/10 = 0.001, MOI PR8 = 0.01), measured using HA assays (mean with SEM, *N* = 4 experiments, B/Wisc/10 48 and 72 HPI *p = 0.0286, PR8 18, 24, 48, 72 HPI *p = 0.0286). HAU = hemagglutination units, HPI = hours post infection. **d** End point titers for multicycle infections of control and B3GAT1 MDCKs (MOI B/Wisc/10 = 0.001, MOI PR8 = 0.01, 24 HPI), measured by plaque assay (mean with SEM, *N* = 4 experiments, B/Wisc/10 *p = 0.0286, PR8 *p = 0.0286). PFU = plaque-forming units. **e** Enterovirus infections of control and B3GAT1 A549s (24 HPI). Scale bar is 50 μm. **f** Quantification of enterovirus infection (24 HPI) in control or B3GAT1 A549s (mean with SEM, *N* = 4 experiments, 23229 *p = 0.0286, 23087 *p = 0.0286, CVB3 *p* = 0.3429). Percent infection was measured via high-content imaging after staining for viral RNA. **g** TCID50 quantification of EV-D68 infection in control and B3GAT1 A549s (mean with SEM, *N* = 4, 23229 *p = 0.0286, 23087 *p = 0.0286). Statistical analyses were performed using a two-tailed Mann-Whitney U test. * indicates *p* < 0.05, ns = not significant. Source data are provided as a Source Data file.

We next performed single-cycle infections with each virus in the panel in both mCherry and B3GAT1 A549 cells. Strikingly, B3GAT1 overexpression significantly reduced infection by all tested influenza viruses (Fig. 3a, b). We then selected two viruses from the panel, a contemporary IBV and a lab strain of IAV, and performed multi-cycle growth curves in MDCK cells overexpressing human B3GAT1 (Supplementary Fig 4). We observed delayed replication kinetics and reduced endpoint titers for both viruses in B3GAT1-expressing MDCK cells (Fig. 3c, d). To confirm that our findings would translate beyond cancerous cell culture lines, we overexpressed B3GAT1 in NL20 cells, which are non-tumor human lung cells, and again observed suppression of infection with IBV (Supplementary Fig 4). Finally, we sought to determine if B3GAT1-mediated viral restriction could be overwhelmed. To test this, we infected cells across a range of multiplicities of infection (MOIs) and found that while higher MOIs resulted in more total B3GAT1 cells infected, B3GAT1 still restricted infection with respect to control at all MOIs tested (Supplementary Fig. 5).

A respiratory virus unrelated to the influenza viruses that also requires sialic acid for attachment and/or entry is enterovirus D68 (EV-D68)^46^. EV-D68 circulates primarily in children causing mild to severe respiratory disease and, in some cases, acute flaccid myelitis^47^. As there are no currently available vaccines or antivirals for EV-D68, we tested if EV-D68 would be susceptible to B3GAT1 overexpression. Upon infection of B3GAT1 A549 cells with two clinical isolates of EV-D68, we observed a significant reduction in infection as measured by positive double-stranded RNA staining (Fig. 3e, f). In contrast, infection with a different enterovirus, coxsackievirus B3 (CVB3), which utilizes the coxsackievirus and adenovirus receptor protein for entry^48^, was unaffected (Fig. 3e, f). Further, EV-D68 infection of B3GAT1-expressing cells resulted in a ~100-fold reduction in viral infection as determined by TCID50 (Fig. 3g). Finally, to verify that EV-D68 infection was blocked at the binding and entry stage, we transfected genomic RNA and did not detect an observable difference in levels of viral replication in mCherry- and B3GAT1-overexpressing cells (Supplementary Fig. 6).

### B3GAT1 can be safely expressed in vivo to protect against lethal influenza virus infection

**Fig. 4:**
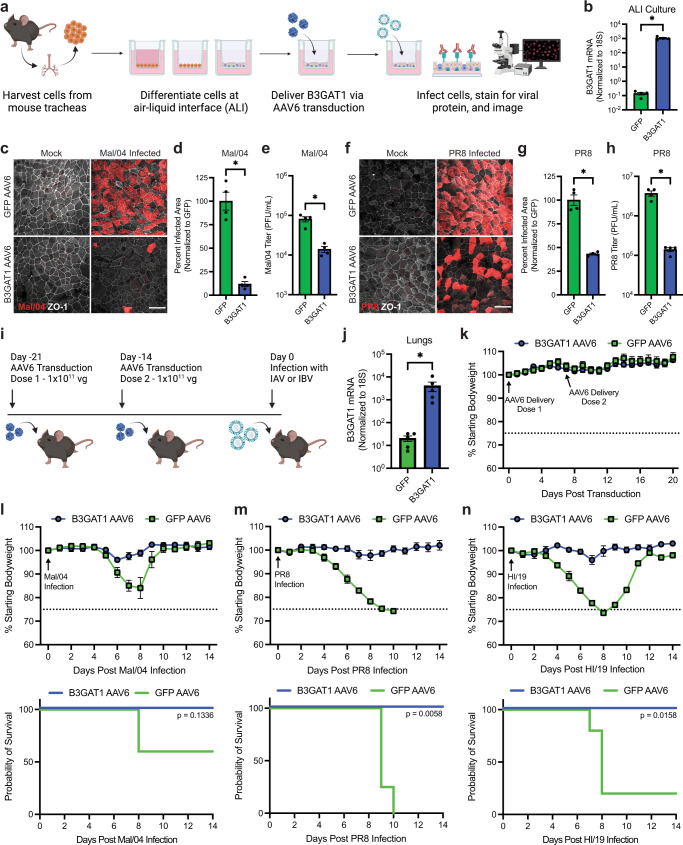
B3GAT1 can be safely expressed in vivo to protect against lethal influenza virus infection. **a** Schematic depicting the generation and transduction of murine tracheal epithelial cell cultures at air–liquid interface (ALI). AAV = adeno-associated virus. **b** Expression of B3GAT1 in GFP AAV6 and B3GAT1 AAV6 transduced ALI cultures, measured by qRT-PCR (mean with SEM, *N* = 4 independent cultures). **c** Representative images of B/Malaysia/2506/2004 (Mal/04) or mock-infected ALI cultures (MOI = 1, 24 HPI). Scale bar is 30 µm. **d** Quantification of the infected area of ALI cultures from Fig. 4c (mean with SEM, *N* = 4 independent cultures, *p = 0.0286). **e** End point titer of Mal/04 infections in ALI cultures (MOI = 1, 24 HPI), measured by plaque assays (mean with SEM, *N* = 4 independent cultures, *p = 0.0286). PFU = plaque-forming units. **f** Representative images of PR8 or mock-infected ALI cultures (MOI = 10, 24 HPI). Scale bar is 30 µm. **g** Quantification of the infected area of ALI cultures from Fig. 4f (mean with SEM, *N* = 4 independent cultures, *p = 0.0286). **h** End point titer of PR8 infections in ALI cultures (MOI = 10, 24 HPI), measured by plaque assays (mean with SEM, *N* = 4 independent cultures, *p = 0.0286). **i** Timeline for the AAV transduction and influenza virus challenge of C57BL/6J mice. vg = viral genomes. **j** qRT-PCR for B3GAT1 RNA in mouse lung homogenates (25 days post transduction, mean with SEM, *N* = 5 mice, *p = 0.0079). **k** Bodyweight of mice following B3GAT1 AAV6 and GFP AAV6 transduction (*N* = 5 mice). **l** Bodyweight (top) and survival (bottom) of B3GAT1 AAV6 and GFP AAV6 transduced mice following Mal/04 infection (50,000 PFU, *N* = 5 mice). **m** Bodyweight (top) and survival (bottom) of B3GAT1 AAV6 and GFP AAV6 transduced mice following PR8 infection (100 PFU, *N* = 4 mice). **n** Bodyweight (top) and survival (bottom) of B3GAT1 AAV6 and GFP AAV6 transduced mice following A/Hawaii/70/2019 (HI/19) infection (30,000 PFU, *N* = 5 mice). Dotted line on all bodyweight plots depicts 75% bodyweight humane endpoint. All bodyweight plots show the mean with SEM. Unless otherwise stated, statistical analyses were performed using a two-tailed Mann-Whitney U test. Survival plots *p* values were determined using the logrank (Mantel–Cox) test. * indicates *p* < 0.05. Source data are provided as a Source Data file.

Since B3GAT1 overexpression induced strong and broad protection from viral infection, we next wanted to evaluate the potential of B3GAT1 expression as an antiviral strategy. We, therefore, expanded our analysis to primary, differentiated respiratory epithelial cells by generating air–liquid interface (ALI) cultures from murine tracheas (Fig. 4a). Staining for ciliated cells confirmed that the ALI cultures were differentiated at the time of transduction (Supplementary Fig 7). Utilizing serotype 6 adeno-associated viral (AAV6) gene transfer vectors, we then delivered human B3GAT1 to the ALI cultures and observed an ~10,000 fold increase in B3GAT1 RNA compared to GFP-transduced ALI cultures (Fig. 4a, b). B3GAT1 overexpression in these AAV-transduced ALI cultures reduced the infection and productive titer of both the IBV strain B/Malaysia/2506/2004 (Mal/04) (Fig. 4c–e) and the IAV strain A/Puerto Rico/8/1934 (PR8) (Fig. 4f–h).

As B3GAT1 overexpression had provided protection in all tested cell culturing systems, we next wanted to determine if we could utilize this strategy to protect from influenza disease progression in vivo. AAV6 vectors were used to deliver human B3GAT1 (which is highly conserved between humans and mice) or GFP to female C57BL/6J mice via intranasal inoculation (Fig. 4i). Delivery of transgenes to the respiratory epithelium using this method was confirmed via qRT-PCR for B3GAT1 mRNA in mouse lung homogenates and microscopy of lung sections (Fig. 4j, Supplementary Fig 8) and mice transduced with B3GAT1 did not exhibit any detectable clinical symptoms, suggesting that transient upregulation of B3GAT1 is well-tolerated in vivo (Fig. 4k). As a first test of the potential of B3GAT1 expression as an antiviral strategy in vivo, we infected mice with Mal/04 (a mouse-adapted IBV) and found that exogenous B3GAT1 expression in the respiratory tract greatly improved infection outcome (Fig. 4l). We next wanted to test a broader range of B3GAT1 antiviral activity; we therefore infected with a highly mouse-adapted H1N1 IAV (PR8) and A/Hawaii/70/2019 (HI/2019), a contemporary non-mouse adapted pandemic-like H1N1 IAV. We found that while both viruses caused severe, lethal disease in our GFP-transduced control mice, mice transduced with B3GAT1 displayed only minimal bodyweight loss after infection and 100% of the animals survived the challenge (Fig. 4m, n). Finally, quantifying lung viral titers after PR8 infection also showed a small reduction in titer in B3GAT1-transduced mice (Supplementary Fig. 8). These data together provide proof-of-concept that B3GAT1 upregulation has practical utility not only in vitro, but as an in vivo antiviral strategy as well.

## Discussion

In this work, we describe a genome-wide CRISPR activation screen with an IBV and identify B3GAT1 as a potent restriction factor. Mechanistic studies determined that overexpression of B3GAT1 limits the amount of sialic acid available on the surface of cells by adding terminal GlcA moieties, thereby restricting IBV entry. We further show that B3GAT1-mediated sialic acid replacement broadly restricts viruses that require sialic acid for entry, including IAVs, IBVs, and EV-D68. Expression of B3GAT1 in primary respiratory epithelial cultures as well as in mice effectively restricted influenza disease, demonstrating that the B3GAT1 phenotype is not limited to cell culture systems. Inducing the expression of B3GAT1 may therefore represent an attractive candidate for the development of broadly acting host-directed antiviral therapeutics.

In previous work, host-directed antiviral prophylactics and therapeutics have relied on small-molecule inhibitors or treatment with recombinant proteins to non-specifically activate the immune system^9^. This report demonstrates that the transient overexpression of a delivered host factor is capable of functioning as a broad-spectrum, prophylactic, antiviral strategy in vivo. Despite this advancement, numerous challenges to translate this observation into clinical use remain. Perhaps the most obvious is the mechanism of B3GAT1 induction. While we have utilized AAV vectors to deliver B3GAT1, this approach is tailored more appropriately to gene therapy applications and their clinical use for the prevention and treatment of an acute infectious disease is unlikely. This is in no small part due to the length of time required for maximal expression of the transgene and the potential immune responses against the vector itself^49–52^. To overcome this limitation, emerging state-of-the-art genetic technologies such as mRNA-based gene delivery and CRISPR-mediated gene upregulation may provide a practical path forward^53–56^.

Irrespective of the gene delivery or modulation approaches, the parameters of in vivo gene expression must also be more completely defined. For example, future studies will be needed to determine the minimal proportion of cells in the respiratory tract that need to express B3GAT1 to confer protection against viral infection. Additionally, the magnitude of B3GAT1 upregulation and the time required for the targeted cells to be sufficiently depleted of sialic acid in vivo also remain unknown. It is also important to note that the targeted induction of a specific host factor like B3GAT1 is not mutually exclusive with other proposed antiviral strategies. Perhaps the most relevant to the current study is DAS181, a bacterial sialidase in clinical trials that specifically cleaves sialic acid in the respiratory tract to treat influenza virus infection^57^. We anticipate that DAS181 treatment could be especially effective in combination with B3GAT1 and potentially speed up the time necessary for turnover of surface sialylated glycans after initiation of B3GAT1 overexpression. Another example is the mRNA delivery of Cas13a-based therapeutics to target the viral genome directly^53,58^, which could be multiplexed with host gene modulation approaches. Even further, targeting multiple host restriction or dependency factors simultaneously may allow for more complete protection from infection.

Another important consideration of this and other potential host-directed therapeutic strategies is the likelihood of toxicity in the host. While we did not observe any obvious morbidities resulting from B3GAT1 overexpression in mice during our treatment period, it remains unknown if B3GAT1 overexpression or the resulting depletion in sialic acid has any effect on lung function. Indeed, sialic acid is hypothesized to promote lung hydration and epithelial barrier integrity by generating an anionic shield in the respiratory epithelium^59^ and can even serve an antiviral role when added to secreted mucins^60^. Further, in basal conditions, B3GAT1 is exclusively expressed in subsets of cells in the nervous system and in mature natural killer cells^61,62^. Since B3GAT1 is not normally expressed in respiratory tissues, the effects of GlcA/HNK-1 glycan surface expression in the lungs remain to be determined. Finally, the overexpression of B3GAT1 may have different effects in mice compared to other mammalian systems. Thus, future studies to evaluate the effects of B3GAT1 overexpression on global lung physiology and/or the immune system in multiple pre-clinical models are important next steps.

In summary, we identified B3GAT1 as a broadly acting viral restriction factor amenable to targeted host-directed antiviral strategies. Our data show that infection with IBVs and IAVs is significantly hindered by B3GAT1 both in vitro and in vivo, and that viral restriction occurred irrespective of when the virus was isolated or the specifics of its receptor preferences. Further, our experiments with EV-D68 suggest that the entry of many, if not all, viruses that require sialic acid for entry would also be inhibited. Other viruses not specifically tested in this study but which we predict could be susceptible to B3GAT1 activity include important human pathogens such as the human coronaviruses OC43 and HKU-1^63^, parainfluenza virus types 1 and 3^64,65^ and Mumps virus^66^. While much work remains before the clinical use of host-directed antiviral therapies, these data suggest that continued research in this area may eventually lead to novel and potentially truly broad-spectrum antivirals.

## Methods

### Ethics statement

All procedures involving laboratory mice were approved by the Duke University IACUC under the protocol numbers A189-18-08 and A142-21-07. Mice were housed with up to five mice per cage and the ambient room conditions ranged from 70–74 °F and 30–70% humidity with a 12-hour dark/light cycle. Animals were assessed daily after infection and a 75% starting bodyweight cutoff was used as a humane endpoint. Primary euthanasia was typically performed via CO_2_ asphyxiation, followed by bilateral thoracotemy as the secondary method.

### Viruses

Influenza virus strains were obtained from BEI Resources, the International Reagent Resource, the American Type Culture Collection (ATCC), or were kindly provided by Dr. Peter Palese. For A/Philippines/2/1982, a reassortment virus with HA/NA segments from A/Philippines/2/1982 and the 6 internal segments from A/Puerto Rico/8/1934 was used. B/Yamagata/16/1988 PB1-mNeon was generated as described previously^39,67^. Influenza virus was propagated either on MDCK cells or in embryonated chicken eggs at 37 °C (IAV) or 33 °C (IBV) for 48–72 h. To verify each strain, viral RNA was isolated using TRIzol reagent (Invitrogen 15596026) and ~1 kb of segments 4, 6, and either 3 or 5 were amplified using the SuperScript III One-Step RT-PCR System (Invitrogen 12574-035) and sequenced. Reference sequences were unable to be located for A/Scotland/840/1974. For A/Scotland/840/1974, BLAST analysis identified the closest matching reference sequences to be A/Albany/20/1974 for segment 4 (CY021093.1), segment 5 (CY021096.1), and segment 6 (CY021095.1). In addition to influenza virus, experiments were performed with coxsackievirus B3-RD (CVB3) and two enterovirus D68 isolates (USA/MD/2009-23229 and USA/CO/2018-23087). CVB3 was provided by Jeffrey Bergelson and originally obtained by the ATCC. Enterovirus D68 isolates were provided by the Center for Disease Control and Prevention (CDC). These viral strains were propagated in HeLa cells and purified by ultracentrifugation over a 30% sucrose cushion, as described previously^68^. Purity of viral stocks was confirmed by Sanger sequencing of VP1 using enterovirus-specific primers, as described previously^69^.

### Plasmids

To generate a CRISPR activation cell line, lenti dCAS9-VP64_Blast (Addgene 61425) was used to package dCas9-VP64 for lentiviral overexpression. For hit validation, individual sgRNAs for B4GALNT2 (CAGGTGCAGGACGCTTGGGA), B3GAT1 (GGACCCGAGCTGCTAATGGT), NXPE4 (TTATTAGCTCAAGCAGAGGT), TGM2 (CCCAGGGGACCGGAGCCCGA), ANXA11 (GAGCAGCAGCCTTCCGGCTG), GPHA2 (AAGAGCAAGATCCCCCAGGT), or a non-targeting sgRNA (AAAACAGGACGATGTGCGGC) were cloned into the pXPR_502 vector (Addgene 96923). To generate cell lines that ectopically overexpress B3GAT1, B3GAT1 full-length ORF cDNA was obtained from transOMIC (transOMIC DQ893324) and cloned into the pLEX vector for lentiviral overexpression. The AAV packaging plasmids pTRs-eGFP, pXX6-80, and pXR6 were kind gifts from Dr. Aravind Asokan. To generate AAVs for B3GAT1 overexpression, the human B3GAT1 ORF was cloned into the pTRs AAV genomic plasmid.

### Cell lines

A549, HEK 293T, MDCK, and NL20 cell lines were obtained from the ATCC and cultured at 37 °C with 5% CO_2_. A549 and HEK 293T cells were grown in Dulbecco’s Modified Eagle Medium (DMEM) with 5–10% FBS, GlutaMAX, and penicillin/streptomycin. MDCK cells were grown in Minimal Essential Medium (MEM) with 5% FBS, GlutaMAX, sodium bicarbonate, HEPES, and penicillin/streptomycin. A549, HEK 293T, and MDCK media were supplemented with 2.5 µg/mL Plasmocin. NL20 cells were grown in Ham’s F-12 medium with 4% FBS, 1.5 g/L sodium bicarbonate, 2.7 g/L glucose, MEM nonessential amino acids, 0.005 mg/mL insulin, 10 ng/mL EGF, 0.001 mg/mL transferrin, 500 ng/mL hydrocortisone, and penicillin/streptomycin. HeLa cells (clone 7B, derived from parental line HeLa S3, ATCC) were provided by Jeffrey Bergelson, Children’s Hospital of Philadelphia, Philadelphia, PA, and cultured in MEM supplemented with 5% FBS, non-essential amino acids, and penicillin/streptomycin. Lentiviruses were packaged as described previously^12^ and cell lines were transduced in complete media containing 0.001% DEAE dextran. Post-transduction, cells were selected for in puromycin (A549s = 1 µg/mL, MDCKs = 5 µg/mL, NL20s = 0.3–0.5 µg/mL) or blasticidin (A549s = 1.6 µg/mL).

### Murine tracheal epithelial cell cultures at air–liquid interface (ALI)

Tracheas were collected from female wild-type C57BL/6J mice and tracheal epithelial cells were harvested, then differentiated following a previously published protocol^70^ with slight modifications. Briefly, tracheas were digested in pronase (Roche) and washed to collect epithelial cells. Washes were passed through a 70 µm strainer and the collected cells were treated with DNase I (Sigma). Fibroblasts were eliminated by incubating cells in mTEC basic media with 10% FBS on Primaria plates (Corning) for 3–4 h at 37 °C. Nonadherent cells were collected and seeded at a density of 100,000 cells/cm^2^ onto transwell inserts (Corning 3470) pre-coated with rat tail collagen I (Corning). Cells were grown in mTEC/Plus media freshly supplemented with 50 nM retinoic acid (Sigma R2625) and 10 µM Y-27632 (Tocris 1254) for 3 days. To differentiate cultures, media was removed from the apical chamber to establish an air–liquid interface, and media in the basal chamber was replaced with mTEC/SF supplemented with 50 nM retinoic acid. Cultures were differentiated for at least 2 weeks before use in experiments. To make mTEC basic medium, a 1:1 vol/vol mixture of DMEM and Ham’s F-12 was prepared and supplemented with 15 mM HEPES, 4 mM GlutaMAX, 3.6 mM sodium bicarbonate, and penicillin/streptomycin. mTEC/Plus and mTEC/SF were prepared from mTEC basic media as previously described^70^. For all ALI experiments, *n* refers to cultures independently generated from genetically distinct mice.

### CRISPR activation screen

Cells transduced with dCas9-VP64 (A549-dCas9 VP64) were selected and isolated to generate a stable clonal cell line. Two replicates each containing 5 × 10^7^ A549-dCas9 VP64 cells were transduced with lentiviruses encoding the Calabrese sgRNA library set A (Addgene 92379) at an MOI of 0.5. After transduction, cells were expanded and selected with puromycin and blasticidin for 5 days. Half the library was then collected for each replicate, cells were lysed with DNA/RNA shield (Zymo R1100) and lysates were stored at −80 °C. The remaining ~1 × 10^8^ cells per replicate were infected with B/Yamagata/16/1988 at an MOI of 5 and incubated for 24 h in post-infection media (OptiMEM, 0.4% BSA, 0.01% FBS) supplemented with TPCK-treated trypsin (Thermo 20233) at a concentration of 0.2 µg/mL to facilitate multi-cycle infection. Media was then replaced with DMEM supplemented with 10% FBS, GlutaMAX, and penicillin/streptomycin and cells were incubated at 33 °C for 48 h to allow sufficient cytopathic effect from infection. Cells were then trypsinized, replated to remove dead cells, and allowed to outgrow at 33 °C. Upon outgrowth (~1 × 10^8^ cells/replicate), half of the cells were pelleted and lysed for gDNA collection with DNA/RNA shield and the remaining cells (~5 × 10^7^ cells/replicate) were infected again with B/Yamagata/16/1988 at an MOI of 5. This process of outgrowth, gDNA collection, and re-infection was repeated an additional time for three total rounds of infection until both replicate populations did not exhibit obvious cytopathic effects following infection. Genomic DNA was then isolated using QIAamp DNA Blood Maxi Kit (QIAGEN 51194). 18.5 µg of genomic DNA was used as a template for PCR using Ex Taq DNA polymerase (Takara RR001). PCR products were resolved using electrophoresis on a 3% agarose gel and extracted using the GeneJet gel extraction kit (Thermo K0692). Library quality was assessed and PCR products were quantified on a bioanalyzer using the Agilent DNA 1000 kit (Agilent 5067-1504). Libraries were then sequenced on an Illumina MiSeq. After sequencing, fastq files were extracted, raw reads were mapped and normalized, and genes were ranked using the MAGeCK pipeline^38^. Raw sequencing data from the screen will be made available upon publication.

### Influenza virus infections

For IAV and IBV infections in A549, MDCK, and NL20 cell lines, samples were incubated with virus diluted in PBS/BSA infection media (PBS, 0.4% BSA, Mg^2+^/Ca^2+^, penicillin/streptomycin) for 30 min (NL20) or 1 h (A549, MDCK) at 37 °C (IAV) or 33 °C (IBV) unless otherwise noted. Virus was then removed and replaced with either complete growth media (single cycle) or post-infection media (OptiMEM, 0.35% BSA, 0.01% FBS, penicillin/streptomycin) supplemented with TPCK-treated trypsin (Thermo 20233) at a concentration of 0.2 µg/mL (A549s) or 1 µg/mL (MDCKs) (multicycle). For single-cycle infections using the multi-strain influenza virus panel, the IAVs A/WSN/1933 and A/Scotland/840/1974 were incubated at 33 °C to facilitate high-throughput reading of the panel plates. To evaluate sialic acid receptor preference, wild-type A549s were treated with 50 mU/mL sialidase S (Agilent GK80021) or no sialidase in A549 growth medium for 2.5 h at 37 °C, then washed twice with PBS to remove all sialidase S enzyme prior to infection. For infection of murine tracheal epithelial ALI cultures, membranes were washed with PBS to clear away mucus, then apically infected with virus diluted in PBS/BSA for 1 h at 37 °C (IAV) or 33 °C (IBV). Cells were washed basally and apically with PBS three times to clear the unbound virus. Fresh mTEC/SF was added to the basal chamber, and cultures were returned to incubate at air–liquid interface overnight. To determine viral titer from ALI cultures, apically released virus was collected at the indicated time points by incubating in 200 µL PBS for 1 h at 37 °C or 33 °C for analysis via plaque assay.

### Enterovirus infections

B3GAT1 and mCherry A549 cells were seeded on poly-lysine treated eight-well chamber slides (Sigma S6815) for confocal microscopy or 24 well plates for high-content image quantification. The following day, cells were infected with the indicated viruses, incubated at 33 °C (EV-D68) or 37 °C (CVB-3) for 24 h, then processed for immunofluorescence microscopy. For TCID50 assays, B3GAT1 and mCherry A549 cells seeded in 96-well plates were infected with serial dilutions of the indicated viral stocks, with the 10^−2^ dilution equivalent to ~10^6^ PFUs. Cells were infected at 33 °C (EV-D68) or 37 °C (CVB3) for 48 h and then stained with crystal violet. For viral RNA transfections, viral RNA was isolated from infected HeLa cells 12 h following infection with the indicated virus. Cells were lysed and total RNA was prepared using the Sigma GenElute total mammalian RNA miniprep kit, according to the manufacturer’s protocol. B3GAT1 or mCherry A549 cells were reverse transfected with 1 μg of viral RNA using Dharmafect-1 transfection reagent (Dharmacon) according to the manufacturer’s instructions.

### Flow cytometry

All cells were resuspended in PBS+ 1% BSA prior to analysis. Infected NL20 cells and cells incubated with fluorescent infectious virus were fixed in 2% formaldehyde in PBS for at least 10 min at room temperature prior to flow analysis. Flow cytometry was performed using a BD Biosciences FACS Canto II Flow Cytometry System and BD FACSDiva software. When necessary, compensation was calculated and applied to eliminate signal bleed-through. Data were analyzed using FlowJo software. Gating strategies for quantification of flow cytometry experiments in Figs. 1e, 2c, i, and Supplementary Fig 4c are demonstrated in Supplementary Fig 9.

### Immunofluorescence microscopy

To visualize B3GAT1 subcellular localization, indicated cell lines were labeled with CellLight Golgi GFP BacMam 2.0 reagent (Fisher C10592). 48 h after plating, cells were fixed with 4% formaldehyde in PBS for 15 min at room temperature and then washed with PBS. Cells were permeabilized with 0.1% Saponin in PBS for 15 min at room temperature then blocked in PBS + 5% BSA + 0.1% Saponin + 0.1% Tween-20 for 1 h at room temperature. Samples were stained overnight with anti-B3GAT1 primary antibody (Sigma HPA069468, 1:100) in PBS + 0.5% BSA + 0.1% Saponin + 0.1% Tween-20. Samples were then stained with Alexa-Fluor conjugated secondary antibody (1:1000) in PBS + 0.5% BSA + 0.1% Saponin + 0.1% Tween-20 for 1 h at room temperature. Cells were washed to remove secondary antibody, then incubated with Hoechst 33342 to stain nuclei. Slides were mounted using Prolong Diamond antifade mountant and imaged on a Zeiss 780 Upright Confocal microscope. For cells infected with influenza virus that were read via high-content imaging, fixed cells were blocked with 5% BSA in PBS + 0.1% Triton X-100 (0.1% PBS-Triton) for 1 h at room temperature. Cells infected with the IAV/IBV panel were stained with CR9114 anti-HA antibody (Creative Biolabs, PABX-119, 1 µg/mL) overnight at 4 °C followed by anti-human Alexa Fluor 488 antibody (1:1000) for 1 h at room temperature in 0.1% PBS-Triton + 0.5% BSA. For the overwhelming B3GAT1 experiment, cells infected with B/Yamagata/16/1988 were stained with CR9114 as above. Cells infected with A/Puerto Rico/8/1934 were stained with sera collected from A/Puerto Rico/8/1934-infected mice (1:500) followed by anti-mouse Alexa Fluor 488 antibody (1:1000) for 1 h at room temperature in 0.1% PBS-Triton +0.5% BSA. Nuclei were stained for 20 min using Hoescht 33342 (1:2500) diluted in PBS, and cells were imaged on the CX5-Cell Insight plate reader. Murine ALI cultures were fixed in 2% formaldehyde in PBS for 15 min at room temperature, after which membranes were extracted from transwells, blocked with 3% BSA in PBS + 0.2% Triton X-100 (0.2% PBS-Triton) for 1 h at room temperature, and incubated with primary antibody diluted in blocking buffer overnight at 4 °C. Cells were stained for AcTub (clone EPR16772, Abcam ab179484, 1:1000), ZO-1 (Invitrogen 61-7300, 1:25), and for viral protein using sera collected from A/Puerto Rico/8/1934 or B/Malaysia/2506/2004 infected mice (1:250). Membranes were washed with 0.2% PBS-Triton, then stained with Alexa-Fluor secondary antibodies (1:1000) diluted in blocking buffer for 1 h at room temperature. After washing again with 0.2% PBS-Triton, nuclei were stained with 1:2000 Hoechst 33342 dye for 20 min at room temperature. Membranes were mounted on slides with Prolong Diamond antifade mountant and pressed with small magnets for at least 16 h prior to imaging on a Zeiss 780 Upright Confocal microscope. For enterovirus infections, cells were fixed in 4% PFA, then incubated in 0.25% Triton X-100 to permeabilize cell membranes for a minimum of 15 min at room temperature. Cells were incubated with primary antibody (at a 1:200–1000 dilution) for 1 h at room temperature, followed by washing three times in PBS. A recombinant α-dsRNA antibody ( J2, provided by Abraham Brass, University of Massachusetts, Kerafast ES2001)^71^ was used to visualize viral RNA (1:100). To visualize the enterovirus VP1 protein, a rabbit polyclonal antibody specific for EV-D68 (Genetex GTX132313) was used (1:1000). Cells were then incubated for 30 min at room temperature with Alexa-Fluor secondary antibodies. Samples on slides for confocal microscopy were washed, mounted with Vectashield (Vector Laboratories) containing 4′,6-diamidino-2-phenylindole (DAPI), and imaged on a Zeiss 780 Upright Confocal microscope. For quantification of enterovirus infection, stained samples on 24 well plates were incubated with DAPI solution to stain nuclei and cells were imaged on the CX5-Cell Insight plate reader.

### Image quantification

For high-content imaging on the CX5 CellInsight, 27 unbiased images were captured across 3 wells for each condition and percent infection was averaged. For imaging ALI membranes, 3 images were captured for each membrane. Total fluorescent infected area was determined in FIJI by setting thresholds and using the analyze particles function. The three quantified images were then averaged for the relevant conditions to determine percent infected area for each independently generated ALI culture.

### Fluorescent staining of cell surface glycans

Indicated cell lines were seeded on glass coverslips coated with poly-lysine solution and incubated overnight at 37 °C. Cells were then fixed with 4% formaldehyde in PBS for 15 min at room temperature and washed with PBS. Samples were stained with 20 µg/mL WGA (Vector Laboratories, FL-1021-5), MAL I (Vector Laboratories, FL-1311), SNA (Vector Laboratories, FL-1301), or GNL (Vector Laboratories, FL-1241) fluorescent lectins in PBS for 1 h at room temperature. For staining of HNK-1/GlcA, cells were blocked for 1 h at room temperature with PBS + 5% BSA, then incubated with anti-HNK-1 primary antibody (Sigma C6680, 1:250) overnight at 4 °C in PBS + 0.5% BSA. Cells were washed and incubated with anti-mouse Alexa Fluor 488 antibody for 1 h at room temperature. All samples were washed and stained with Hoechst 33342 (Thermo H3570) to visualize nuclei, then mounted using Prolong Diamond Antifade (Thermo P36965).

### Hemagglutination and plaque assays

For hemagglutination assays, serial 1:2 dilutions of virus were prepared in cold PBS in a V-bottom 96-well plate. An equivalent volume of 1:40 diluted chicken or turkey blood was added to the diluted virus, and plates were incubated at 4 °C overnight before analysis. Results are expressed in HA units, defined as the reciprocal of the highest dilution at which hemagglutination occurs. If there was no apparent hemagglutination, samples were assigned a value of 0.5 HAU. For plaque assays, confluent MDCK cells were infected with tenfold virus dilutions in a six-well plate at 37 °C (IAVs) or 33 °C (IBVs) for 1 h. Virus was then removed and an agar overlay (MEM, GlutaMAX, sodium bicarbonate, HEPES, penicillin/streptomycin, BSA, DEAE dextran, 0.5% Oxoid agar (Thermo LP0028)) with 1 µg/mL TPCK-treated trypsin (Thermo 20233) was poured over cells before returning plates to the incubator. When plaques were visible (48–72 h post infection), cells were fixed by applying 4% formaldehyde in PBS to the top of the agar overlay for at least 3 h at room temperature. The agar overlay was removed, and cells were stained with 1:2000 primary antibody either overnight at 4 °C or for 2–3 h at room temperature. A/Puerto Rico/8/1934 was stained using sera from A/Puerto Rico/8/1934 infected mice, while B/Malaysia/2506/2004 and B/Wisconsin/1/2010 were stained using sera from B/Malaysia/2506/2004 infected mice. Cells were then incubated with secondary anti-mouse HRP (VWR NXA931V, 1:4000) for at least 1 h at room temperature, and stained plaques were visualized with KPL TrueBlue substrate. Antibodies were diluted in 5% nonfat milk in PBS + 0.05% Tween-20.

### Viral binding assay

Approximately 80 mL of B/Yamagata/16/1988 virus in egg allantoic fluid was fluorescently labeled with 80 µL of Vybrant^TM^ DiO Cell-Labeling Solution (ThermoFisher, V22886) for at least 1 h at room temperature. Virus was then concentrated using ultracentrifugation with a 30% sucrose cushion for 1 h at 110,405 xg on the Sorvall TH-641 swinging bucket rotor to remove residual DiO labeling reagent. Viral pellets were resuspended in 1 mL PBS and saved at 4 °C until use. Indicated cell lines were trypsinized, neutralized with DMEM + 5% FBS to create a cell suspension, and divided into at least two technical replicates for each experiment. Cells were then washed once with PBS + 1% BSA and incubated with fluorescent B/Yamagata/16/1988 virus in PBS/BSA infection media for 1 h on ice. Following incubation, cells were pelleted, viral inoculum was decanted, and cells were fixed with 2% formaldehyde in PBS at room temperature for 15 min. Fixed cells were washed with PBS + 1 % BSA and analyzed via flow cytometry.

### qRT-PCR

RNA was extracted from cultured cells using the Monarch Total RNA Miniprep Kit (NEB T2010). For RNA prepared from mouse lung homogenate, whole mouse lungs were collected and homogenized in PBS. Total lung RNA was then extracted from 100 uL of homogenate using TRIzol reagent (Invitrogen 15596026). Quantitative RT-PCR (qRT-PCR) was performed using the EXPRESS One-Step Superscript qRT-PCR kit (Invitrogen 11781200) on an Applied Biosystems QuantStudio 3 Real-Time PCR System. mRNA was detected using commercial TaqMan probes for B3GAT1 (Hs01024500_m1), B4GALNT2 (Hs00963127_m1), NXPE4 (Hs00916016_m1), TGM2 (Hs01096680_m1), ANXA11, (Hs01012624_g1) or GPHA2 (Hs00369982_g1) and normalized to endogenous 18S RNA (Applied Biosystems 4318839).

### Western blotting

Indicated cell lines were lysed by pelleting 3 × 10^6^ cells, resuspending in 200 µL lysis buffer (50 mM Tris pH 7.4, 150 mM NaCl, 1 mM EDTA, 1% Triton X-100), and incubating for 10 min at room temperature. Supernatant was combined with Laemmli sample buffer and 5% 2-beta mercaptoethanol and heated at 95 °C for 5 min. Lysate was then loaded onto a 4–20% Mini-PROTEAN TGX Stain-Free Gel (Bio-Rad). After running samples, stain-free gels were imaged on a Bio-Rad ChemiDoc Imaging System, and protein was transferred to a nitrocellulose blotting membrane (GE 10600002) for 60 min at 60 V. Membranes were blocked in 5% nonfat dry milk in PBS + 0.1% Tween-20 (0.1% PBS-T) for 1–2 h at 4 °C, then incubated overnight at 4 °C in anti-B3GAT1 antibody (Sigma AMAB91575, 1:1000) diluted in blocking buffer. Membranes were washed with 0.1% PBS-T, incubated in goat anti-mouse HRP secondary (Invitrogen A16072, 1:5000) for 1 h at room temperature, and washed again with 0.1% PBS-T. To develop blots, membranes were incubated in Clarity Western ECL substrate (Bio-Rad) and exposed to film.

### Release of N-linked glycans

Approximately 5 × 10^7^ of both B3GAT1 and mCherry A549 cells were washed with PBS and collected via scaping, pelleted, and frozen. Cell pellets were resuspended in 500 µL of 50 mM ammonium bicarbonate and passed ten times through a 23-gauge syringe to homogenize. Cells were lysed by sonication for 2 min and 30 s at 15 s intervals, and lysate was heated at 95–100 °C for 5 min. Samples were then denatured by adding 10 µL denaturing buffer (NEB PNGaseF kit, P0709) and incubating at 50 °C for 30 min. To desalt, samples were added to 10 kDa cutoff spin filters pre-washed with 50 mM ammonium bicarbonate and centrifuged at 14,000 × *g* for 15 min. After washing with 50 mM ammonium bicarbonate, samples were removed from the spin filters and resuspended in 500 µL 50 mM ammonium bicarbonate. To release N-linked glycans, samples were treated with PNGaseF (NEB, P0709) for 20 h at 37 °C. Released N-linked glycans were centrifuged in pre-washed 10 kDa cutoff spin filters, purified by passage through a C18 cartridge, and lyophilized prior to further analysis.

### MALDI-TOF analysis of N-linked glycans

Lyophilized samples were permethylated by reconstituting in DMSO and treating with methyl iodide on DMSO/NaOH mixture. After quenching the reaction with water, dichloromethane was used to extract the reaction mixture. Permethylated glycans were dried and reconstituted in 20 µL methanol, and 2 µL of reconstituted sample was combined with 2 µL DHB matrix. This mixture was spotted on a MALDI plate and analyzed on the AB Sciex TOF/TOF 5800 System Mass Spectrometer using a laser intensity of 6400, reflector mode, and positive ion mode. Mass spectra diagrams were generated using AB Sciex Data Explorer v4.5. Clusters of evenly spaced peaks differing by 32 Da were noted for all peaks corresponding to masses of glycans containing glucuronic acid. This Δ−32Da mass shift was assumed to be the loss of a methoxy group located on the 4th carbon of the per-O-methylated glucuronic acid, a reaction that is known to occur due to exposure of the uronic acid ethers to basic conditions^72^. In each cluster, the peaks spaced 32 Da apart from one another were considered to originate from the same glycan. MALDI-TOF glycan spectra were confirmed using MS/MS. Percent composition was determined by comparing the intensity peak for each glycan mass. For glycan masses containing glucuronic acid, relative compositions were determined assuming that (1) the Δ−32Da mass shifts correspond to the loss of a methoxy group and (2) the summation of each of the peaks in each cluster series can be taken to represent the theoretical intensity of the original peak without any degradation.

### Monosaccharide composition analysis

Monosaccharide composition analysis was performed by combined GC-MS of the per-*O*-trimethylsilyl (TMS) derivatives of the monosaccharide methyl glycosides produced from the sample by acidic methanolysis as described previously^73^. Briefly, released N-linked glycans were heated with methanolic HCl in a sealed screw-top glass test tube for 18 h at 80 °C. After cooling and removal of the solvent under a stream of nitrogen, the samples were re-N-acetylated at room temperature using methanol: pyridine: acetic anhydride (2:1:1) and dried again. The sample was then derivatized with Tri-Sil® (Pierce) at 80 °C for 20 min. GC/MS analysis of the TMS methyl glycosides was performed on an Agilent 7890A GC interfaced to a 5975 C MSD, using an Supelco Equity-1 fused silica capillary column (30 m × 0.25 mm ID). Inositol was added to the samples as an internal standard prior to data collection.

### Sialic acid linkage analysis

Tandem mass spectrometry (MS/MS) was used to characterize the sialic acid linkages of N-linked glycans, according to previously published protocols^43^. Briefly, lyophilized permethylated, sialylated, N-linked glycans were reconstituted in 200 µL of a 50:50 MeOH:H_2_O mixture with 1 mM Li_2_CO_3_ and analyzed on a Thermo Orbitrap Fusion Tribrid Mass Spectrometer using ion trap mode. Fragment ions diagnostic of specific linkages were annotated based on previously published methods^43^.

### Adeno-associated virus (AAV) production

Production and purification of AAVs were adapted from refs. 74,75,76. Briefly, AAVs were produced by transfecting HEK 293 T cells with pXX6–80, pTRs-B3GAT1 or pTRs-eGFP, and pXR6 in a 2:1:1 ratio (by µg amount). Transfected cells were incubated at 37 °C for 96 h to allow AAVs to assemble. To harvest AAVs, media was removed and cells were collected using a cell scraper. AAVs in the media were harvested via precipitation in 0.5 M NaCl/8% PEG-8000 at 4 °C overnight. Intracellular AAVs were extracted by thermal lysis of the harvested cells followed by chemical lysis using 0.5% sodium deoxycholate. Cell lysates were clarified and passed through 0.22 µm filters. AAVs were purified by applying clarified cell lysates to heparin columns (Cytiva 17040701). Eluted AAVs were transferred to 100,000 MWCO dialysis tubing (Millipore Z726060) and dialyzed against pharmaceutical-grade PBS overnight at 4 °C. AAV stocks were concentrated using Amicon centrifugal concentration units (Millipore UFC901024). The titer of each AAV stock was determined via qPCR, as previously demonstrated^77^. Additionally, transduction efficiencies of each AAV stock were validated by transducing A549 cells followed by detection of either sialic acids via fluorescein-labeled WGA (Vector Laboratories FL-1021) or HNK-1 via immunostaining (Sigma C6680).

### AAV transductions

Differentiated murine ALI cultures were washed with PBS to clear mucus, then simultaneously transduced on the apical and basal side with GFP AAV6 or B3GAT1 AAV6. Apical cells were incubated in 50 µL AAVs in mTEC basic (MOI = 100,000) for 8 h at 37 °C, while basal cells were incubated in 500 µL AAVs in mTEC/SF (MOI = 100,000) for 48 h at 37 °C. After 7 days, cells were transduced a second time following the same protocol. Experiments were performed 7–8 days after the second transduction. For AAV deliveries in vivo, 9–13-week-old female wild-type C57BL/6J mice were anesthetized with 80 µL of ketamine/xylazine mixture. GFP AAV6 and B3GAT1 AAV6 were diluted in pharmaceutical-grade PBS and delivered intranasally at a dose of 10^11^ viral genome equivalents/mouse. Mice were positioned upright with their mouths held closed to increase efficiency of the delivery^78^. Mice were transduced twice to maximize transgene expression^79^, with 7 days between deliveries, and all influenza virus infections occurred 14 days after the second transduction. Mice were monitored after transduction to assess the effects of B3GAT1 AAV6 and GFP AAV6 on animal health. To confirm transduction efficiency, mice 21 days post transduction were perfused intracardially with 12 mL of PBS and lungs were inflated intratracheally with 1 mL of a 1:1 mix of OCT compound and 8% formaldehyde in PBS. Inflated lungs were collected and fixed overnight in 4% formaldehyde in PBS at 4 °C. The following day, lungs were transferred to 30% sucrose in PBS and incubated at least 24 h at 4 °C before cryosectioning. 8 µm cryosections were taken, adhered to glass slides, dried, and outlined with a hydrophobic pen to facilitate staining. Re-hydrated sections were stained with Hoechst 33342 and mounted under a glass coverslip using Prolong Diamond Antifade mountant. Images were captured on a Zeiss 780 Upright Confocal microscope.

### Mouse infections with influenza virus

AAV-transduced mice were anesthetized with 80 µL of ketamine/xylazine mixture prior to infection. B/Malaysia/2506/2004, A/Puerto Rico/8/1934, and A/Hawaii/70/2019 were diluted in pharmaceutical grade PBS and administered intranasally in 40 µL total volume. After infection, bodyweights were obtained daily and mice were euthanized upon reaching the humane threshold as detailed in the ethics statement. Percent starting bodyweights were rounded to the nearest whole percent to determine if a mouse reached the humane threshold. For lung viral titering, whole mouse lungs were homogenized in PBS and lung titer was determined via plaque assay.

### Statistics and reproducibility

Statistical tests used to determine significance are indicated in the figure legends. For all null hypothesis testing with the Mann-Whitney U test, a confidence interval of 95% was used to assess significance. Values not detected due to technical limitations of an assay were excluded from subsequent statistical analyses. Error bars represent standard error of the mean for all graphs. Unless otherwise noted, a minimum of three technical replicates were conducted for each independently conducted experiment. Technical replicates were not conducted for experiments using genetically distinct mice or independently generated ALI cultures. Statistical analyses and graphs were generated using Prism 9 software. All experiments depicted by representative images were performed independently at least twice with similar results.

### Reporting summary

Further information on research design is available in the Nature Research Reporting Summary linked to this article.

## Supplementary information

Supplementary Information

Description of Additional Supplementary Files

Supplementary Data 1

Supplementary Data 2

Supplementary Data 3

Reporting Summary

## Data Availability

The raw sequencing data generated in this study have been deposited at NCBI GEO under the accession number GSE205009 and are publicly available. Source data for all graphs are provided as a Source Data file. Source data are provided with this paper.

## Source data

Source Data
