# Supplementary material for: Cellular glycan modification by B3GAT1 broadly restricts influenza virus infection
Source: Nat Commun. 2022 Oct 29;13:6456. doi: 10.1038/s41467-022-34111-0 (PMC9617049; doi:10.1038/s41467-022-34111-0)
Supplement: Supplementary file 1 — Supplementary Information [file 41467_2022_34111_MOESM1_ESM.pdf]

Supplementary information, Trimarco and Nelson et al.

Supplementary Dataset 1. Normalized sgRNA read counts from the CRISPR activation screen. Related to **Fig 1b**.

Supplementary Dataset 2. MAGeCK gene rank analysis of the CRISPR activation screen. Gene rank analysis was completed on normalized reads after the third round of infection with respect to the input replicates as a control. Related to **Fig 1c**.

Supplementary Dataset 3. Quantification of MALDI-TOF peaks of N-linked glycans released from mCherry or B3GAT1 A549 cells. Related to **Fig 2e** and **Fig 2f**.

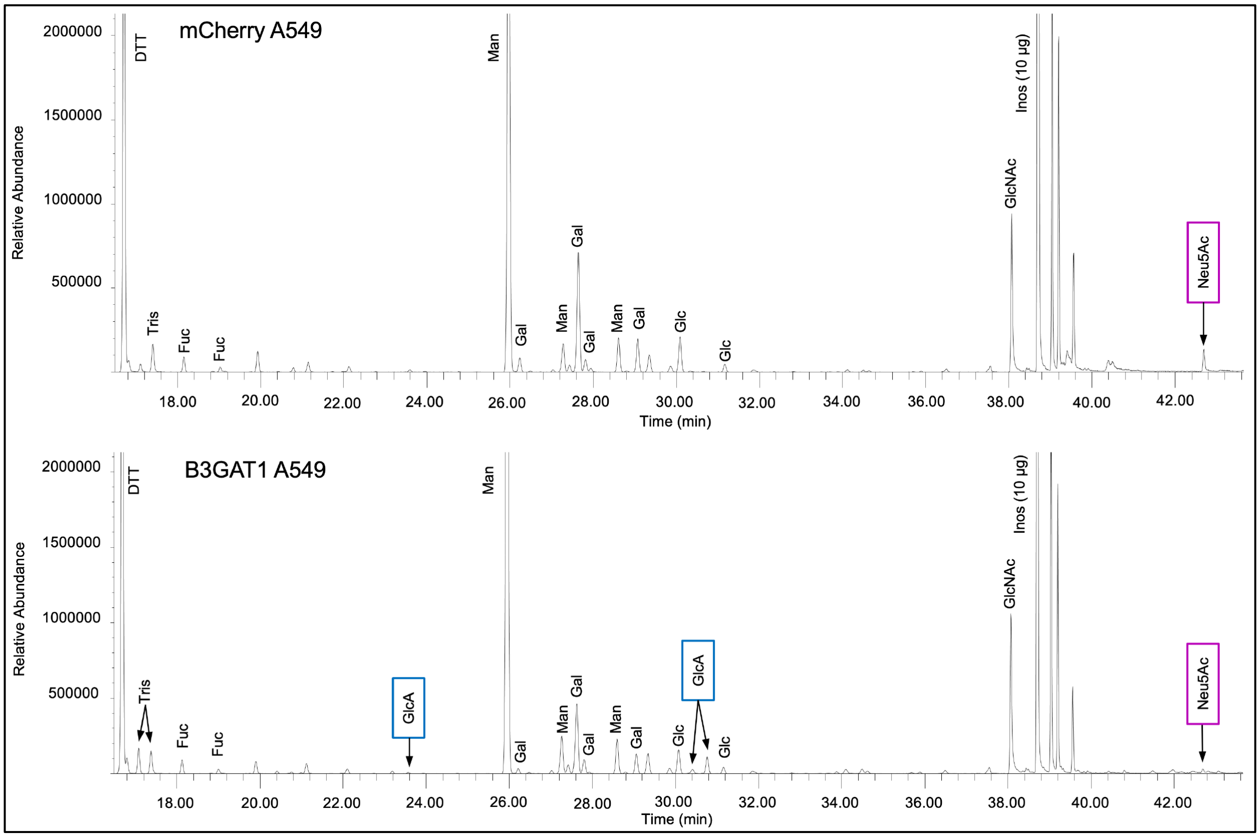

Supplementary Figure 1. Compositional monosaccharide analysis of B3GAT1 A549 cells by GC-MS. Monosaccharides components were derived from N-linked glycans released from mCherry- (top) or B3GAT1- (bottom) overexpressing cells, subjected to gas chromatography and peaks were determined by mass spectrometry. Fuc = fucose, GlcA = glucuronic acid, Man = mannose, Gal = galactose, Glc = glucose, GlcNAc = N-Acetylglucosamine, Inos = inositol (spiked control), Neu5Ac = N-Acetylneuraminic acid. GlcA signatures are denoted with blue boxes, Neu5Ac signatures are denoted with magenta boxes.

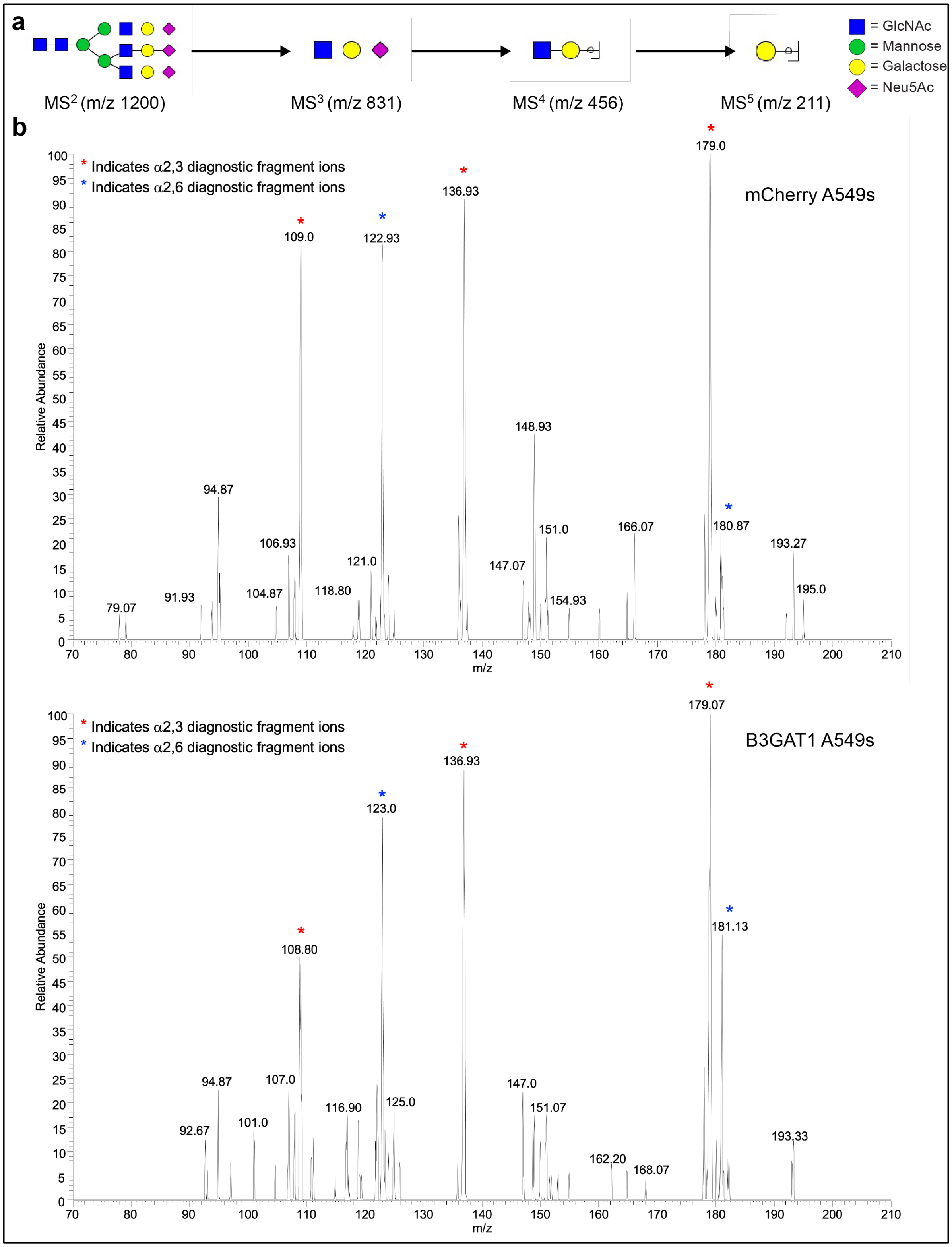

Supplementary Figure 2. Sialic acid linkage analysis of B3GAT1 A549 cells. (a) Fragmentation pathway conducted for sialylated glycans to analyze sialic acid linkage by

tandem mass spectrometry. (b) Spectrum of sialylated glycans released from mCherry A549 cells (top) or B3GAT1 A549 cells (bottom). Fragment ions at m/z ~109 (red asterisk) and ~137 (red asterisk) are diagnostic for α2,3 linkage. Fragment ion at m/z ~123 (blue asterisk) is diagnostic for α2,6 linkage. Fragment ions at m/z ~179 and ~181 appear for both linkages, though a higher relative amount of m/z ~179 (red asterisk) is diagnostic for α2,3 linkage and higher relative amount of m/z ~181 is diagnostic for α2,6 linkage (blue asterisk).

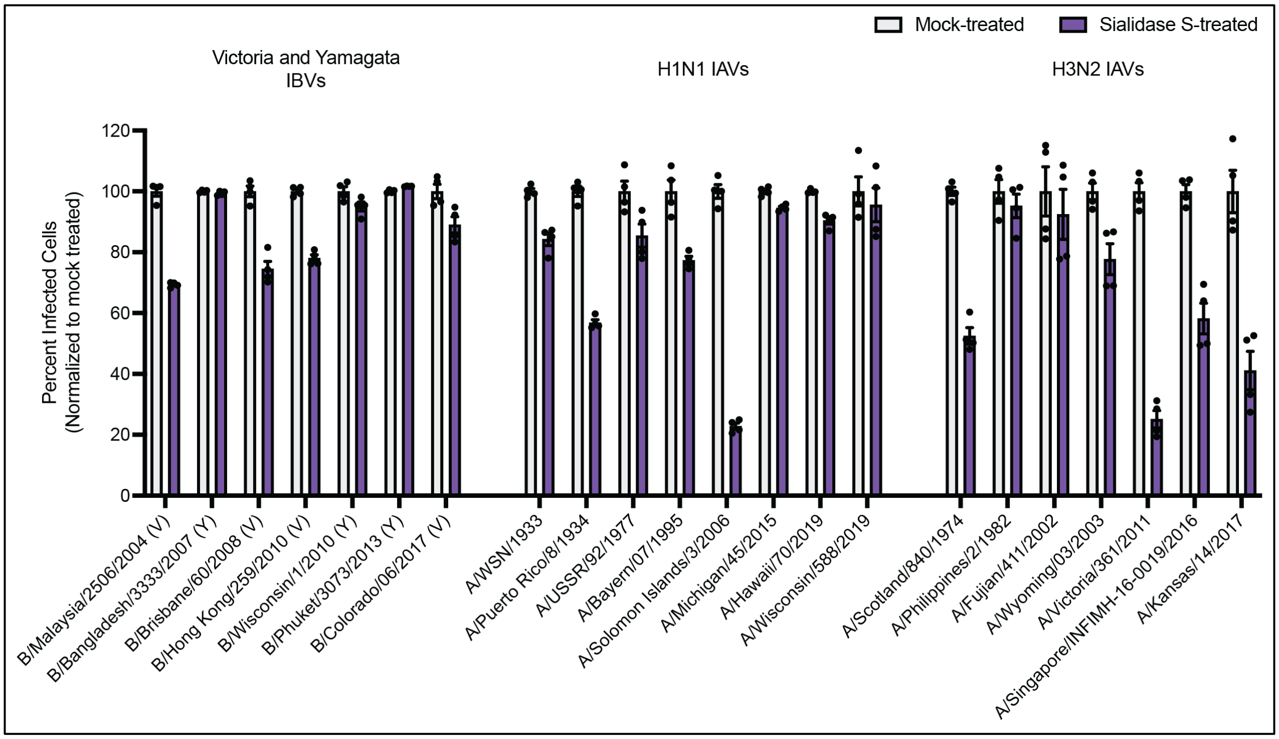

Supplementary Figure 3. Evaluation of viral receptor preference in the influenza virus panel. Single cycle infections (24 HPI) of Sialidase S-treated or mock-treated A549 cells with a panel of influenza viruses. Sialidase S specifically cleaves α2,3-linked sialic acid. Viruses are ordered based on year of isolation. Assays quantified using high-content cell imaging after staining for viral HA protein (mean with SEM, N=4 experiments). V = Victoria-lineage IBV, Y = Yamagata-lineage IBV.

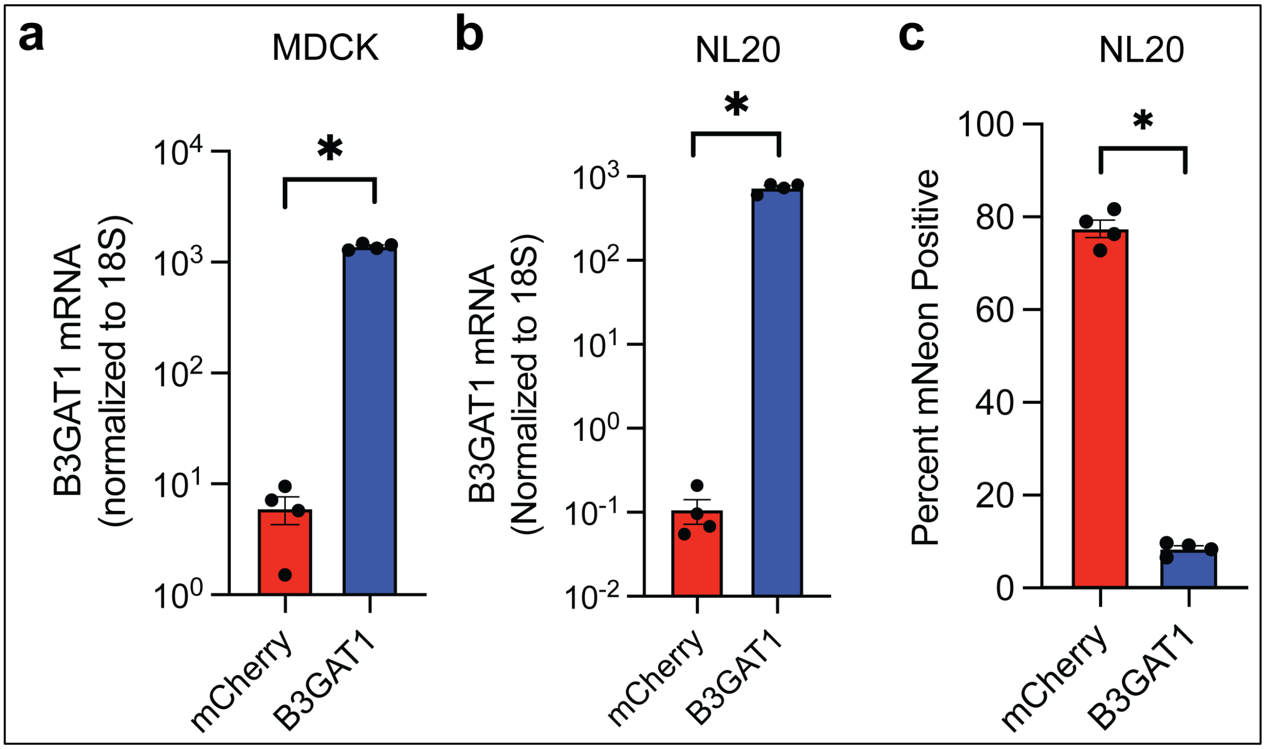

Supplementary Figure 4. Validation of B3GAT1 as an antiviral restriction factor across cell lines. (a) qRT-PCR for B3GAT1 mRNA in the indicated MDCK cell lines (mean with SEM, N=4 experiments, *p = 0.0286). (b) qRT-PCR for B3GAT1 mRNA in the indicated NL20 cell lines (mean with SEM, N=4 experiments, *p = 0.0286). (c) Percent of control or B3GAT1 NL20s infected with Yam/88 PB1-mNeon (MOI=1, 24 HPI, single cycle), measured using flow cytometry (mean with SEM, N=4 experiments, *p = 0.0286). Statistical analyses were performed using the Mann-Whitney U test. * indicates $p < 0.05$.

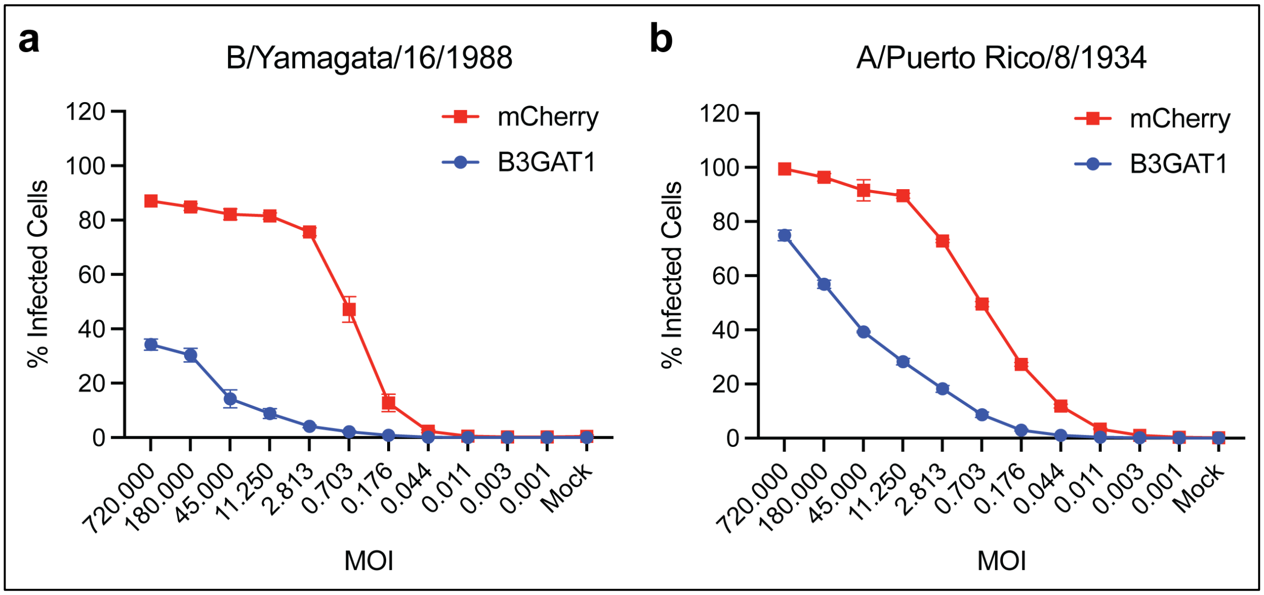

Supplementary Figure 5. The antiviral effect of B3GAT1 overexpression is maintained at high MOI. (a) Percent of control or B3GAT1 A549s infected at the indicated MOIs with B/Yamagata/16/1988 (24 HPI, single cycle, mean with SEM, N=4 experiments). (b) Percent of control or B3GAT1 A549s infected at the indicated MOIs with A/Puerto Rico/8/1934 (24 HPI, single cycle, mean with SEM, N=4 experiments). Percent infected cells measured using high-content cell imaging after staining for viral HA protein.

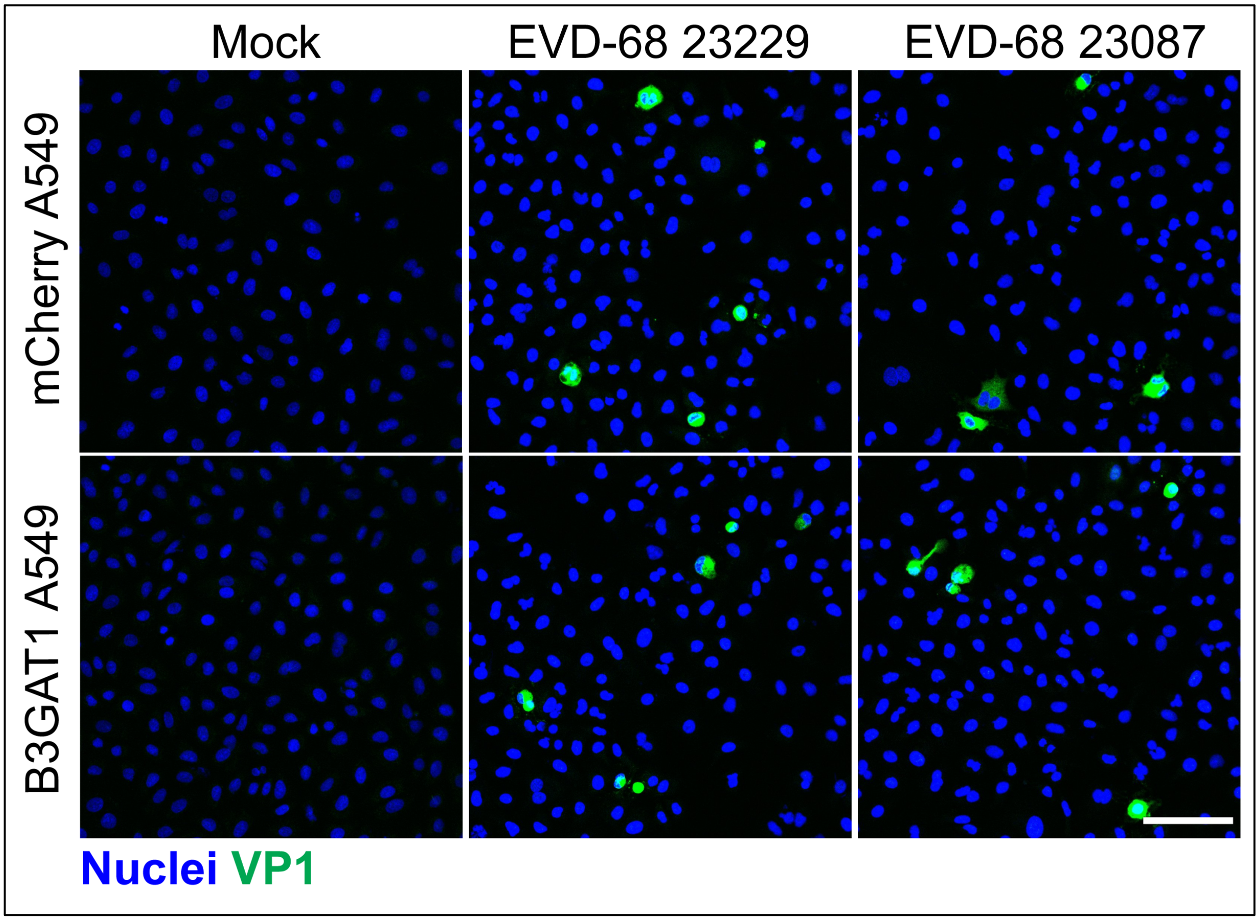

Supplementary Figure 6. B3GAT1 overexpression does not affect enterovirus gene expression at a post-entry step. Immunofluorescent microscopy of enterovirus VP1 expression in B3GAT1 and control A549s transfected with the indicated viral RNA (vRNA). Scale bar is 100 μm.

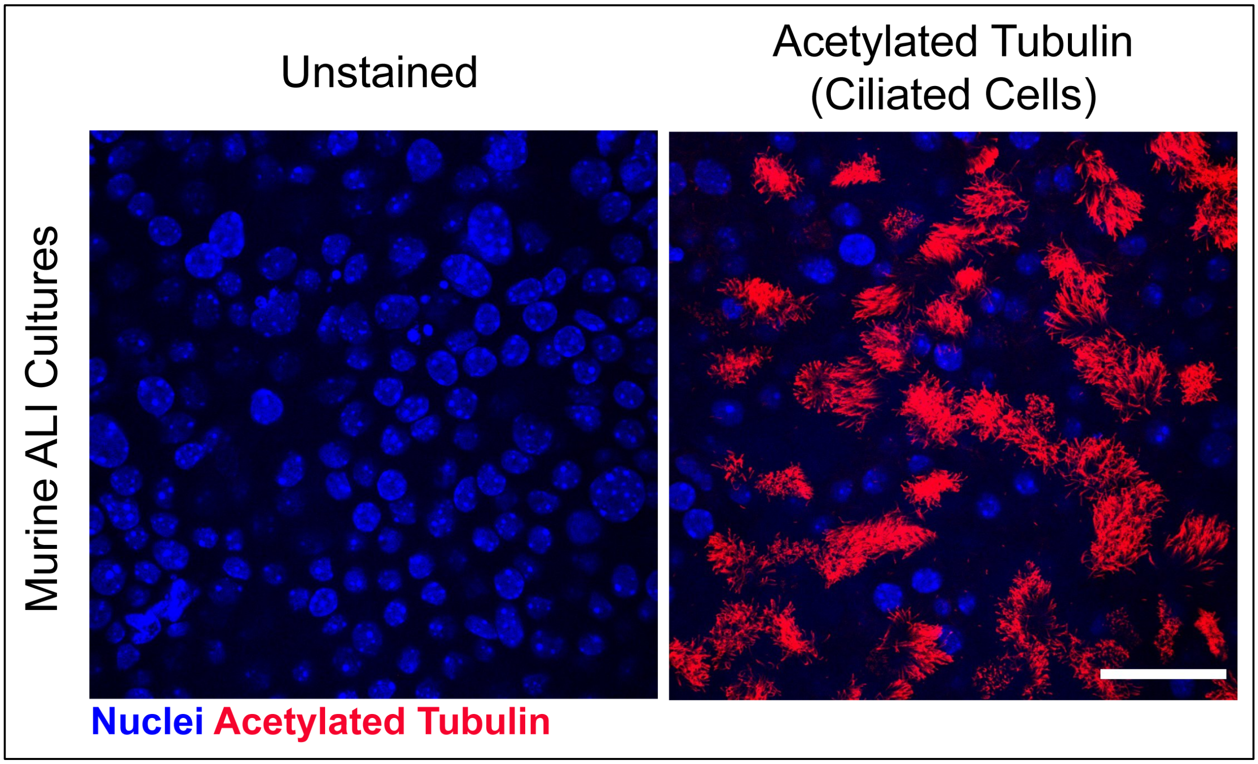

Supplementary Figure 7. Murine ALI cultures display cilia after differentiation. Mouse tracheal epithelial cells were grown at air-liquid interface (ALI) for 2-3 weeks prior to immunofluorescence analysis. Ciliated cells were detected by staining for acetylated alpha tubulin and indicate cellular differentiation. Scale bar is 30 μm.

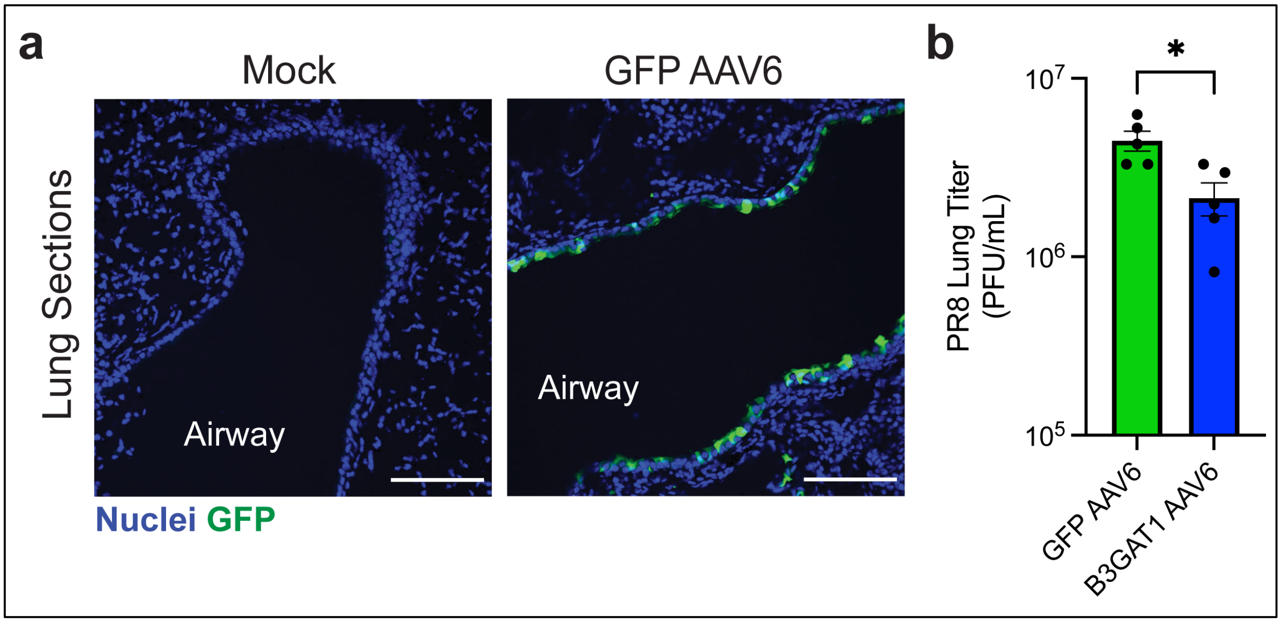

Supplementary Figure 8. Evaluating delivery efficiency and influenza viral titer in AAV-transduced mouse lungs. (a) Distribution of GFP in mouse lung airways at 21 days post-AAV transduction. Scale bars are 100 µm. (b) Viral titer obtained from total lung homogenate from PR8 infected GFP- or B3GAT1-transduced mice (100 PFU infection, 4 DPI, mean with SEM, N = 5 mice, *p = 0.0238). Statistical analysis was performed using the Mann-Whitney U test. * indicates $p < 0.05$.

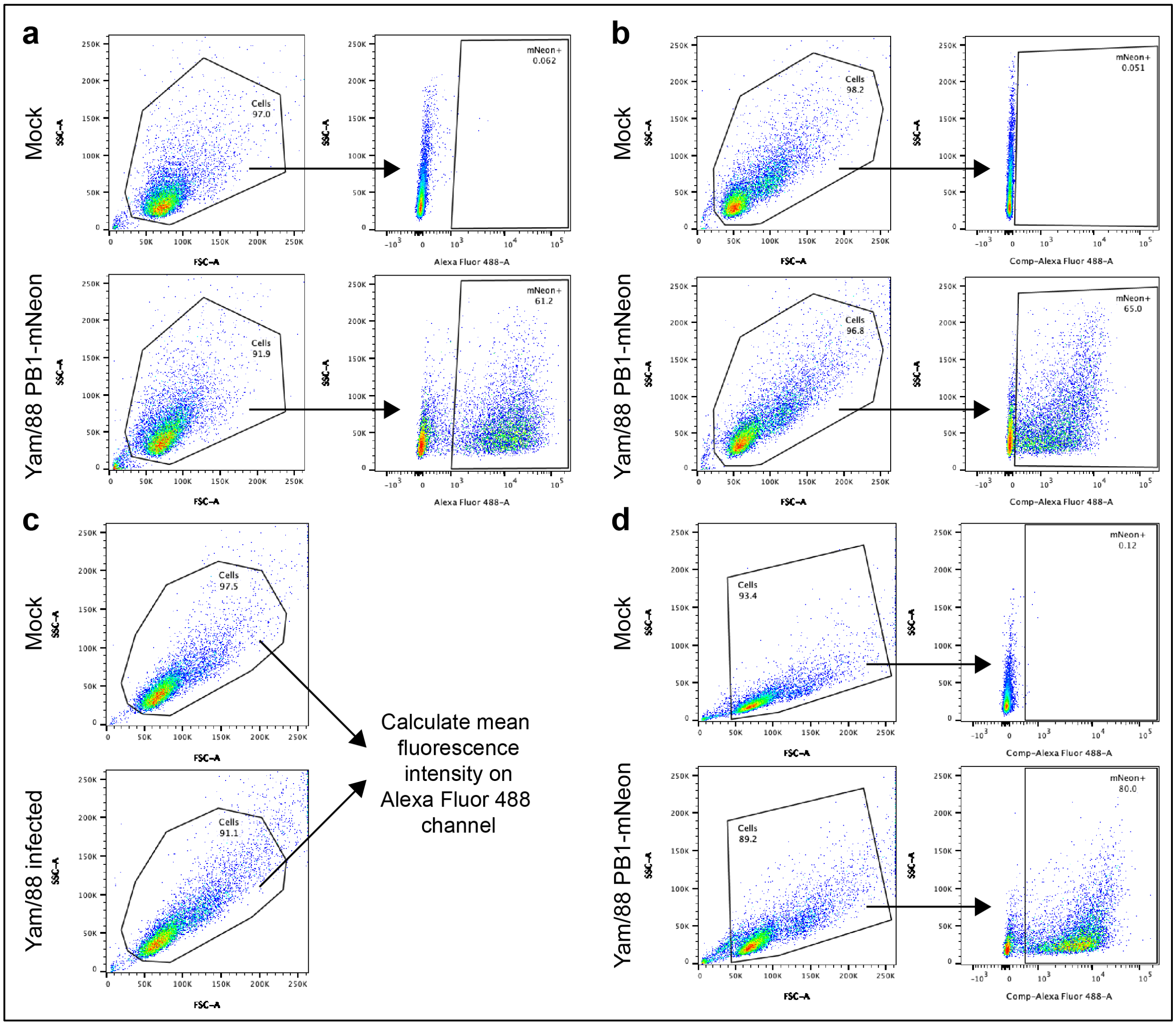

Supplementary Figure 9. Gating strategies for flow cytometry quantification. (a) Gating strategy for mNeon+ population quantification in **Fig 1e** (non-targeting control sgRNA sample shown). (b) Gating strategy for mNeon+ population quantification in **Fig 2c** (mCherry A549 control sample shown). (c) Gating strategy for calculating mean fluorescence intensity in **Fig 2i** (mCherry A549 control sample shown). (d) Gating strategy for mNeon+ population quantification in **Supplementary Figure 4c** (mCherry NL20 control sample shown).
