# Supplementary material for: Cellular glycan modification by B3GAT1 broadly restricts influenza virus infection
Source: Nat Commun. 2022 Oct 29;13:6456. doi: 10.1038/s41467-022-34111-0 (PMC9617049; doi:10.1038/s41467-022-34111-0)
Supplement: Supplementary file 2 — Description of Additional Supplementary Files [file 41467_2022_34111_MOESM2_ESM.pdf]

Description of Additional Supplementary Files

File Name: Supplementary Data 1
Description: Normalized sgRNA read counts from the CRISPR activation screen. Related to Fig 1b.

File Name: Supplementary Data 2
Description: MAGeCK gene rank analysis of the CRISPR activation screen. Gene rank analysis was completed on normalized reads after the third round of infection with respect to the input replicates as a control. Related to Fig 1c.

File Name: Supplementary Data 3
Description: Quantification of MALDI-TOF peaks of N-linked glycans released from mCherry or B3GAT1 A549 cells. Related to Fig 2e and Fig 2f.
