# Supplementary material for: Cellular glycan modification by B3GAT1 broadly restricts influenza virus infection
Source: Nat Commun. 2022 Oct 29;13:6456. doi: 10.1038/s41467-022-34111-0 (PMC9617049; doi:10.1038/s41467-022-34111-0)
Supplement: Supplementary file 6 — Reporting Summary [file 41467_2022_34111_MOESM6_ESM.pdf]

Corresponding author(s): Heaton, NS
Last updated by author(s): Oct 5, 2022

Reporting Summary

Nature Portfolio wishes to improve the reproducibility of the work that we publish. This form provides structure for consistency and transparency in reporting. For further information on Nature Portfolio policies, see our Editorial Policies and the Editorial Policy Checklist.

Statistics

For all statistical analyses, confirm that the following items are present in the figure legend, table legend, main text, or Methods section.

n/a	Confirmed	
☐	☒	The exact sample size (n) for each experimental group/condition, given as a discrete number and unit of measurement
☐	☒	A statement on whether measurements were taken from distinct samples or whether the same sample was measured repeatedly
☐	☒	The statistical test(s) used AND whether they are one- or two-sided<br>*Only common tests should be described solely by name; describe more complex techniques in the Methods section.*
☒	☐	A description of all covariates tested
☒	☐	A description of any assumptions or corrections, such as tests of normality and adjustment for multiple comparisons
☐	☒	A full description of the statistical parameters including central tendency (e.g. means) or other basic estimates (e.g. regression coefficient) AND variation (e.g. standard deviation) or associated estimates of uncertainty (e.g. confidence intervals)
☐	☒	For null hypothesis testing, the test statistic (e.g. F, t, r) with confidence intervals, effect sizes, degrees of freedom and P value noted<br>*Give P values as exact values whenever suitable.*
☒	☐	For Bayesian analysis, information on the choice of priors and Markov chain Monte Carlo settings
☒	☐	For hierarchical and complex designs, identification of the appropriate level for tests and full reporting of outcomes
☒	☐	Estimates of effect sizes (e.g. Cohen's d, Pearson's r), indicating how they were calculated

Our web collection on statistics for biologists contains articles on many of the points above.

Software and code

Policy information about availability of computer code

Data collection	BDFACSDiva was used to collect flow cytometry data.
Data analysis	To analyze the CRISPR activation screen, Illumina BaseSpace was used to demultiplex sequencing reads. MAGeCK (version 0.5.9) was used to map and normalize raw reads, and score gene enrichment after sequencing. FlowJo 10 was used to collect flow cytometry data.

For manuscripts utilizing custom algorithms or software that are central to the research but not yet described in published literature, software must be made available to editors and reviewers. We strongly encourage code deposition in a community repository (e.g. GitHub). See the Nature Portfolio guidelines for submitting code & software for further information.

Data

Policy information about availability of data

All manuscripts must include a data availability statement. This statement should provide the following information, where applicable:
- Accession codes, unique identifiers, or web links for publicly available datasets
- A description of any restrictions on data availability
- For clinical datasets or third party data, please ensure that the statement adheres to our policy

The raw sequencing data generated in this study have been deposited at NCBI GEO under the accession number GSE205009 (https://www.ncbi.nlm.nih.gov/geo/query/acc.cgi?acc=GSE205009) and is publicly available. Source data for all graphs are provided as a Source Data file.

Human research participants

Policy information about studies involving human research participants and Sex and Gender in Research.

Reporting on sex and gender	Human research participants were not involved in this study.
Population characteristics	Human research participants were not involved in this study.
Recruitment	Human research participants were not involved in this study.
Ethics oversight	Human research participants were not involved in this study.

Note that full information on the approval of the study protocol must also be provided in the manuscript.

Field-specific reporting

Please select the one below that is the best fit for your research. If you are not sure, read the appropriate sections before making your selection.

☒ Life sciences ☐ Behavioural & social sciences ☐ Ecological, evolutionary & environmental sciences

For a reference copy of the document with all sections, see nature.com/documents/nr-reporting-summary-flat.pdf

Life sciences study design

All studies must disclose on these points even when the disclosure is negative.

Sample size	No calculations were completed to predetermine sample size. For in vitro experiments, 4 independent experiments were completed to ensure significance could be determined by the non-parametric, two-tailed, Mann-Whitney U test with a 95% confidence interval. Mouse challenge experiments were conducted with 4 or 5 independent animals to ensure p values could be appropriately calculated by the logrank (Mantel-Cox) test based on previous experiments in the laboratory.
Data exclusions	Data were not excluded from analyses except in the case that values were not collected due to technical limitations. Values not detected due to technical limitations of an assay were excluded from subsequent statistical analyses.
Replication	For all statistically analyzed experiments, all data obtained from at least 4 independent, replicate experiments are presented on the final figure graph. For qualitative experiments (such as Western blot, microscopy, etc.), data are representative of at least two independent experiments completed with similar results. For mass spectrometry quantification of glycan composition, experiments were performed once but spectra were confirmed with MS/MS and findings were further supported by preliminary independent glycan UPLC and GC-MS analyses.
Randomization	No specific randomization approach was used. For cell culture experiments, covariates (media, viral inocula, incubator used, etc.) were kept constant within each experiment, and overexpression of a control protein (ie GFP, mCherry) was used to ensure that phenotypes were not due to cellular resources being allocated to protein overexpression. At least four independent experiments are shown on each graph for cell culture experiments to further rule out the effect of covariates on individual experiments. For mouse experiments, covariates (mouse age, housing scheme, etc) were standardized across treatment groups. Mouse experiments were repeated at least twice to further rule out the effect of covariates on individual experiments.
Blinding	Investigators were not blinded to group allocation. Preparation of specific treatments (ie lentiviral and AAV transductions) was necessary to apply to each tested group. To avoid bias in measurements due to absence of blinding, specific measurements were determined by quantitative instruments (flow cytometer, mouse bodyweight scale, etc.) and unbiased imaging/image quantification (CellInsight CX5, FIJI, etc.) when possible.

Reporting for specific materials, systems and methods

We require information from authors about some types of materials, experimental systems and methods used in many studies. Here, indicate whether each material, system or method listed is relevant to your study. If you are not sure if a list item applies to your research, read the appropriate section before selecting a response.

Materials & experimental systems

n/a	Involved in the study
☐	☒ Antibodies
☐	☒ Eukaryotic cell lines
☒	☐ Palaeontology and archaeology
☐	☒ Animals and other organisms
☒	☐ Clinical data
☒	☐ Dual use research of concern

Methods

n/a	Involved in the study
☒	☐ ChIP-seq
☐	☒ Flow cytometry
☒	☐ MRI-based neuroimaging

Antibodies

Antibodies used	PRIMARY ANTIBODIES:<br>Anti-B3GAT1– mouse monoclonal, Sigma AMAB91575, clone CL9499<br>Anti-B3GAT1– rabbit polyclonal, Sigma HPA069468, lot R102639<br>Anti-HNK-1/N-CAM (CD57)– mouse monoclonal, Sigma C6680, clone VC1.1, lot 099M4785V<br>Recombinant anti-influenza B antibody (CR9114) – human monoclonal, Creative Biolabs PABX-119, clone CR9114, lot CB2009Y15<br>Enterovirus anti-dsRNA antibody (J2) – clone J2, obtained from Abraham Brass, University of Massachusetts - Kerafast ES2001<br>Anti-Enterovirus D68 VP1 – rabbit polyclonal, Genetex GTX132313<br>Anti-ZO-1 – rabbit polyclonal, Thermo Scientific 61-7300, lot WC318960<br>Anti-alpha tubulin (acetyl K40) – rabbit monoclonal, Abcam ab179484, clone EPR16772, lot GR3240369-7<br>Mouse serum – sera was collected by our lab from A/Puerto Rico/8/1934 or B/Malaysia/2506/2004 infected mice, and used to stain for viral protein<br><br>SECONDARY ANTIBODIES:<br>Goat anti-mouse Alexa Fluor 488 antibody (Thermo A-11001)<br>Goat anti-mouse Alexa Fluor 594 antibody (Thermo A-11032)<br>Goat anti-rabbit Alexa Fluor 488 antibody (Thermo A-11008)<br>Goat anti-rabbit Alexa Fluor 647 antibody (Thermo A-21245)<br>Goat anti-human Alexa Fluor 488 antibody (Thermo A-11013)<br>Goat anti-mouse HRP secondary (Invitrogen A16072)<br>Goat anti-Rabbit IgG (H+L) Cross-Adsorbed Secondary Antibody, HRP (Thermo A16104)<br>Anti-IgG Sheep Polyclonal Antibody (HRP (Horseradish Peroxidase)) (VWR NXA931)
Validation	All antibodies used in this study obtained from commercial sources were validated by the companies listed in the "Antibodies used" section above. We validate the specificity of the anti-B3GAT1 mouse and rabbit antibodies in Figures 2b and 2d, respectively. Binding of CR9114 antibody to influenza virus hemagglutinin protein is demonstrated in Grover et al. 2014 (PMID: 245038520). Mock-infected staining controls for the CR9114 antibody, J2 antibody, anti-Enterovirus D68 VP1 antibody, and mouse serum are shown in the manuscript to ensure specificity to viral infection components when used for immunofluorescence microscopy. No specific validation for anti-alpha tubulin or anti-ZO-1 was performed in this study.

Eukaryotic cell lines

Policy information about cell lines and Sex and Gender in Research

Cell line source(s)	A549 (ATCC), HEK 293T (ATCC), MDCK (ATCC), NL20 (ATCC), HeLa (provided by Jeffrey Bergelson, Children's Hospital of Philadelphia, Philadelphia, PA - derived from parental line HeLa S3, ATCC).
Authentication	No specific authentication approach was used for cell lines in this study.
Mycoplasma contamination	A549, HEK 293T, and MDCK cells tested negative for mycoplasma contamination. NL20 and HeLa cells were not tested for mycoplasma.
Commonly misidentified lines (See ICLAC register)	No cell lines used in this study are designated as commonly misidentified lines.

Animals and other research organisms

Policy information about studies involving animals; ARRIVE guidelines recommended for reporting animal research, and Sex and Gender in Research

Laboratory animals	Wildtype mice (C57BL/6J) were ordered from Jackson Laboratory. Mice were 9-13 weeks old at time of treatment. Mice were housed with up to 5 mice per cage and the ambient room conditions ranged from 70-74°F and 30-70% humidity with a 12 hour dark/light cycle.
Wild animals	Study did not involve wild animals.

Reporting on sex	All in vivo assays were conducted with female mice.
Field-collected samples	Study did not involve samples collected from the field.
Ethics oversight	All procedures involving laboratory mice were approved by the Duke University IACUC under the protocol numbers A189-18-08 and A142-21-07.

Note that full information on the approval of the study protocol must also be provided in the manuscript.

Flow Cytometry

Plots

Confirm that:

- [x] The axis labels state the marker and fluorochrome used (e.g. CD4-FITC).
- [x] The axis scales are clearly visible. Include numbers along axes only for bottom left plot of group (a 'group' is an analysis of identical markers).
- [x] All plots are contour plots with outliers or pseudocolor plots.
- [x] A numerical value for number of cells or percentage (with statistics) is provided.

Methodology

Sample preparation	Sample preparation is described extensively for each flow cytometry experiment in the methods. Briefly, cells were trypsinized, fixed with 2% formaldehyde when applicable, and ran on a cytometer.
Instrument	BD FACSCanto II
Software	BD FACSDiva, FlowJo 10
Cell population abundance	Cells were not sorted in this study.
Gating strategy	Gating strategies are shown in Supplementary Figure 9.

- [x] Tick this box to confirm that a figure exemplifying the gating strategy is provided in the Supplementary Information.
